# Mechanism-Based Screen Establishes Signalling Framework for DNA Damage-Associated G1 Checkpoint Response

**DOI:** 10.1371/journal.pone.0031627

**Published:** 2012-02-27

**Authors:** Elizabeth Richardson, Simon R. Stockwell, He Li, Wynne Aherne, Maria Emanuela Cuomo, Sibylle Mittnacht

**Affiliations:** 1 Department of Cancer Biology, UCL Cancer Institute, London, United Kingdom; 2 Division of Cancer Biology, The Institute of Cancer Research, London, United Kingdom; 3 Division of Cancer Therapeutics, The Institute of Cancer Research, London, United Kingdom; St. Georges University of London, United Kingdom

## Abstract

DNA damage activates checkpoint controls which block progression of cells through the division cycle. Several different checkpoints exist that control transit at different positions in the cell cycle. A role for checkpoint activation in providing resistance of cells to genotoxic anticancer therapy, including chemotherapy and ionizing radiation, is widely recognized. Although the core molecular functions that execute different damage activated checkpoints are known, the signals that control checkpoint activation are far from understood. We used a kinome-spanning RNA interference screen to delineate signalling required for radiation-mediated retinoblastoma protein activation, the recognized executor of G_1_ checkpoint control. Our results corroborate the involvement of the p53 tumour suppressor (TP53) and its downstream targets p21^CIP1/WAF1^ but infer lack of involvement of canonical double strand break (DSB) recognition known for its role in activating TP53 in damaged cells. Instead our results predict signalling involving the known TP53 phosphorylating kinase PRPK/TP53RK and the JNK/p38MAPK activating kinase STK4/MST1, both hitherto unrecognised for their contribution to DNA damage G1 checkpoint signalling. Our results further predict a network topology whereby induction of p21^CIP1/WAF1^ is required but not sufficient to elicit checkpoint activation. Our experiments document a role of the kinases identified in radiation protection proposing their pharmacological inhibition as a potential strategy to increase radiation sensitivity in proliferating cancer cells.

## Introduction

DNA damage through exposure to ionising radiation (IR) is an important tool in cancer therapy. Radiotherapy features in the treatment of greater than 50% of all cancers and IR is considered the most effective treatment option for inoperable solid tumours [1], [2].

Although objective responses are frequent, long-term remission is not often seen, and patients commonly relapse with tumour re-growth following cessation of treatment [3]. Increasing evidence suggests that the genetic makeup of tumours critically influence the IR-sensitivity of cancer tissue and the duration of remission in therapies involving IR [4]. Loss of either damage repair [5] or damage-inducible cell cycle checkpoint control [6] enhances IR sensitivity, suggesting that both repair efficacy and checkpoint activation confer radioprotection. Other evidence indicates that preferential activation of checkpoint control provides resistance to cancer stem cells [7]. Hence inhibition of repair or checkpoint signalling has been proposed as a strategy for enhancing the response of cancers to radiotherapy [8], [9].

DNA damage-inducible cell cycle checkpoints transiently delay cell cycle progression in proliferating cells, presumably providing time for repair [10], [11]. DNA damage checkpoint control arises at multiple points of the cell cycle including late G_1_ (G1), intra S phase, and the G_2_ phase [12]. Recent years have seen considerable progress in elucidating signalling involved in the different types of checkpoint control. Checkpoint kinases 1 and 2 (CHK1/2) are key executors involved in delaying S and G_2_/M transit [13], [14], [15], [16]. CHKs phosphorylate, and thus inhibit, the dual specificity phosphatases CDC25B and A [17], [18], [19], [20] required for activation of the CDK2 and CDK1 cyclin-dependent kinases which drive DNA synthesis and entry of cells into M phase respectively. Other work demonstrates involvement of MAPKAP-kinase2 (MK2) and MK2-dependent GADD45A biosynthesis [21], [22], and a role for the p53 tumour suppressor protein TP53 in the maintenance of the G2 checkpoint response [23], [24].

G1 checkpoint activation is thought to involve the retinoblastoma tumour-suppressor (RB1) and its paralogues. RB1 inhibits the transcription of gene products required for S phase entry, amongst them the CDK2 activating cyclins E and A [25], and it stabilizes the CDK inhibitory proteins p27^KIP1^/CDKN1B and p21^CIP1/WAF1^/CDKN1A [26]. Exposure of cells to IR leads to accumulation of RB1 in its active, underphosphorylated form [27], [28]. G1 checkpoint activation in irradiated cells is likely to be of dual significance. In response to DNA damage, G1 checkpoint execution may delay progression of G1 cells from entering S phase [29], [30]. G1 checkpoint activation also underlies “adaptation”, which follows escape of damaged cells from G2 arrest [31], [32].

Considerable evidence indicates that RB1 loss favourably affects the response of tumours to radiotherapy. Several clinical studies report that absence of RB1 expression predicts treatment success of therapies involving IR, as indicated by prolonged disease-free survival and absence of distant metastasis [33], [34], [35], [36]. RB1 mediates the proliferation block induced by a range of DNA damaging agents and cells with RB1 loss show accelerated death following DNA damage [29], [37], suggesting that inhibition of radiation-mediated RB1 activation could be a strategy for radio-sensitization of RB1 positive cancers.

The current knowledge as to the signalling that instigates RB1 activation is incomplete and controversial [30], [38], [39], [40]. Here we describe results from a kinome-spanning cell-based screen aimed at the unbiased identification of signalling required for RB1 activation by IR. We identify a group of kinases, hitherto largely unrecognized for their involvement in this context. We characterize the mode by which they interact with the cellular IR response and document their involvement in facilitating G1-arrest and survival of IR-exposed cells.

## Results

### Identification of signalling required for IR–driven RB1 activation

**Figure 1:**
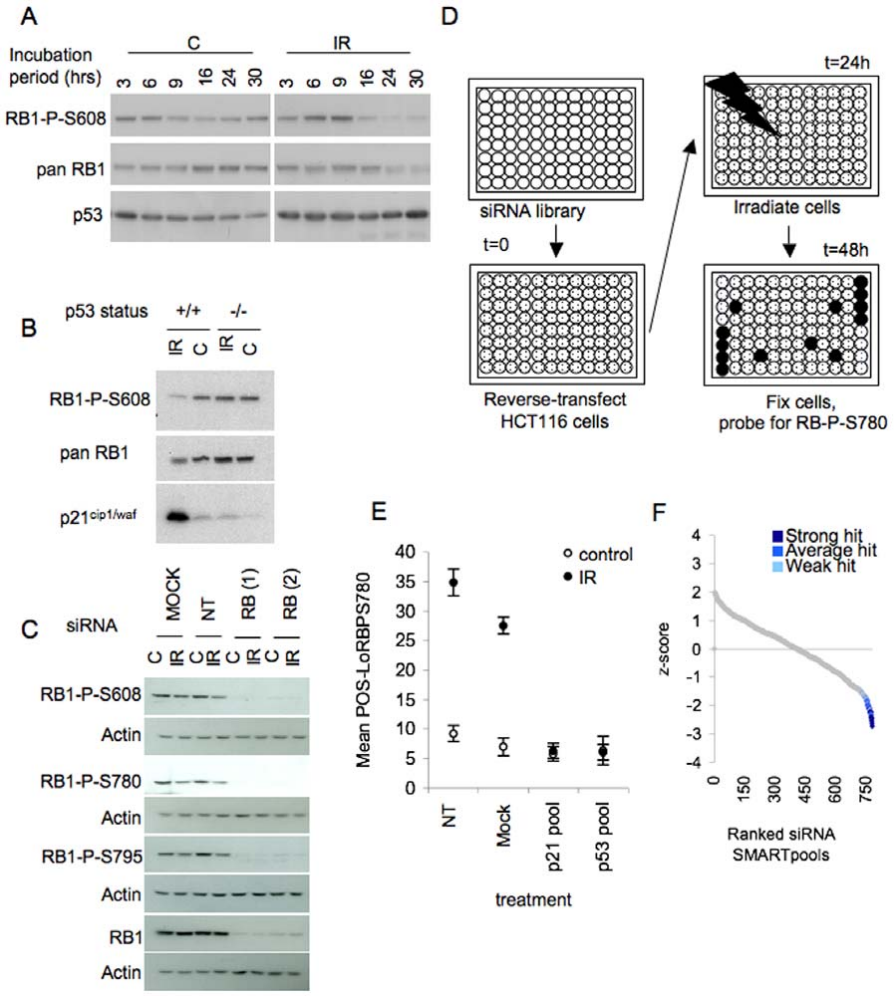
siRNA screen for gene products involved in IR-associated RB1 activation. **A**) **Loss of RB1 phosporylation in IR exposed cells.** HCT116 cells exposed to 5 Gy ionising radiation (IR) or untreated (C) were analysed at the time indicated. Levels of RB1 with phosphorylation on Ser608 (RB1-PS608), total RB1 (RB1) and TP53 were established by immunoblotting. **B**) **Loss of RB1 phosphorylation is TP53-dependent.** TP53 positive (+/+) and isogenic TP53 null (−/−) HCT116 cells exposed to 5 Gy ionizing radiation (IR) or untreated (C) were analysed 24 hours post irradiation. Levels of RB1-PS608, total RB1 and p21^CIP1/WAF1^ were established by immunoblotting. **C**) **IR affects RB1 phosphorylation at multiple sites.** Immunoblots probing for levels of RB1-PS608, RB1-PS780, RB1-PS795 in irradiated (IR) or untreated (C) HCT116. To document RB1 specificity of the signal cells transfected with siRNA duplexes targeting RB1 (RB(1) and RB(2)) or nontargeting control siRNA (NT) were analysed in parallel. Cells were irradiated and harvested at 24 hours following IR. Actin was used as a loading control. **D**) **siRNA screening strategy.** HCT116 were reverse transfected with siRNA library pools in a 96 well format, and irradiated, fixed and stained using anti RB1-PS780 antibody and Hoechst 33342 dye, with timelines as indicated. Plates were analysed using an IN Cell Analyzer 3000 high content platform (GE) with sequential blue and green laser excitation. A set number of cell objects per well were analyzed for nucleus-associated antibody fluorescence (green channel). Hoechst 3342 DNA staining (blue channel) was used for object and compartment identification. Intensity profiles were generated and automatically gated to determine the percentage of cells with sub-normal antibody fluorescence (POS-LoRBPS780) in individual wells. **E**) **Radio-resistant RB1 phosphorylation in cells with siRNA-mediated TP53-signalling knockdown.** Assay set up was as described in D, siRNA pools for TP53, p21^CIP1/WAF1^ or a non-targeting oligonucleotide (nt) were used for transfection. Error bars relate to variance in POS-LoRBPS780 values from triplicate wells. **F**) **Primary screen outcome.** Z-score distribution for target screened. Z-scores were calculated for the mean POS-LoRBPS780 observed in triplicate wells and are plotted in ranked order. Hits are shown colour-coded according to hit class within the Z-score distribution.

To build a screening assay for signalling involved in radiation-mediated RB1 activation we determined the circumstances under which changes in RB1 phosphorylation arise in irradiated cells. We used HCT116 colon-derived carcinoma cells which represent a clinically relevant cancer type for radiation treatment and which express wt RB1. A robust loss of RB1 phosphorylation is seen in these cells between 16 and 24 hours after IR exposure, as indicated by a reduced signal with a phosphorylation-selective antibody for RB1 P-Ser608 (Figure 1A, signal quantification in Figure S1A, A′). This response arose in HCT116 p53+/+ cells but not in isogenic HCT116 p53−/− cells (Figure 1B) for signal quantification see (Figure S1), indicating a critical contribution of TP53 signalling to radiation-mediated RB1 activation. Loss of phosphorylation was seen on other sites, including Ser780 and Ser795 (Figure 1C) and RB1 accumulating in irradiated HCT116 cells had an increased propensity for binding to E2F (Figure S1), indicating general loss of RB1 phosphorylation, and functional activation in these cells.

Using the conditions established we developed a 96-well screening assay (Figure 1D) based on quantitative immunofluorescent detection of phosphorylated RB1 in fixed cells. The assay involved use of an antibody selective for Ser780-phosphorylated (PS780) RB1, which proved technically superior to antibodies for other sites in pilot experiments, and determined the percentage of cells with significantly lower than average signal intensity (POS-LoRBPS780) using high-content single cell-based analysis (see Materials and Methods and Figure S4). Transfection of HCT116 cells with siRNA pools targeting TP53 or its downstream target CDKN1A/p21^CIP1/WAF1^ reduced POS-LoRBPS780 values in these cells more than 6-fold, compared to cells transfected with either a non-targeting siRNA (NT) or without oligonucleotide addition (Mock) (Figure 1E), demonstrating the capacity of the assay to report checkpoint loss following siRNA-based knockdown of relevant signalling components.

To uncover unknown signalling required for IR-mediated RB1 activation we screened an siRNA collection targeting all human kinases and additional accessory molecules (779 targets) involved in phospho-proteome regulation (see Table S1 for list of targets).

Based on mean POS-LoRBS780 values derived from triplicate runs 59 of the 779 targets reached z scores <−1.5 with 22 targets scoring <−2 (Figure 1F). To identify hits with substantial impact we further graded targets scoring with z<−1.5 on the magnitude by which their respective siRNA pools prevented RB1-PS780 loss. ‘Strong’ targets reduced the mean POS-LoRBPS780 by 2-fold or greater, ‘average’ hits led to a reduction of 2- to 1.6-fold and ‘weak’ hits reduced the average POS-LoRBPS780 between 1.6- and 1.4-fold (see Table S1). In total this yielded 41 hits, with 12 scoring ‘strong’, 18 ‘average’ and 11 ‘weak’. In a screen run in parallel using unirradiated cells none of these hits reached z-scores less than −1.3 and the vast majority scored greater −1 (Figure S1), indicating that the observed radiation-resistant RB1 phosphorylation is not due to target knockdown increasing RB1 phosphorylation in unchallenged cells. Gene names, identifiers and screen data for these hits are listed in the Table S1, alongside data for all targets screened.

### Gene ontology and pathway association of hits

Amongst the hits identified was the TP53 target gene CDKN1A/p21^CIP1/WAF1^ [41], predicted from our initial assessment as being critical for RB1 activation, confirming screen performance. The Ataxia telangiectasia mutated (ATM) double stand break (DSB)-activated protein family kinases ATM and ATR, and the checkpoint kinase family kinases CHK1 or CHK2, known to activate TP53 signalling as part of the canonical double stand DNA damage response, did not score, even though these targets were represented in the gene set screened, suggesting that this signalling plays no role in eliciting activation of the checkpoint under investigation. To address if this signalling is indeed unnecessary for RB1 activation following IR we used pharmacological inhibitors for this signalling space (Figure S2). Neither treatment with KU-5593, a selective inhibitor of ATM/ATR, nor the CHK1 selective inhibitor SAR020106 abolished the radiation induced loss of RB1 phosphorylation, (Figure S2). As previously observed (Figure 1), radio-resistant RB1 phosphorylation was seen in parallel samples where cells were transfected with siRNA targeting TP53. Both Ku-5593 and SAR020106 inhibited autocatalytic activity of CHK1 (Figure S2), indicative that they were effective in blocking damage-driven signal transduction in the cell line and at the dose used. Analysis of lysates from cells treated with these inhibitors provided corroborating evidence, revealing net loss of RB1 phosphorylation following IR exposure comparable to that of Mock-treated cells. Together these results corroborate the critical requirement of TP53 and p21^CIP1/WAF1^ as the signal executing axis yet indicate that signalling distinct from the canonical TP53 activating DSB signalling is involved in controlling radiation-mediated RB1 checkpoint activation.

**Figure 2:**
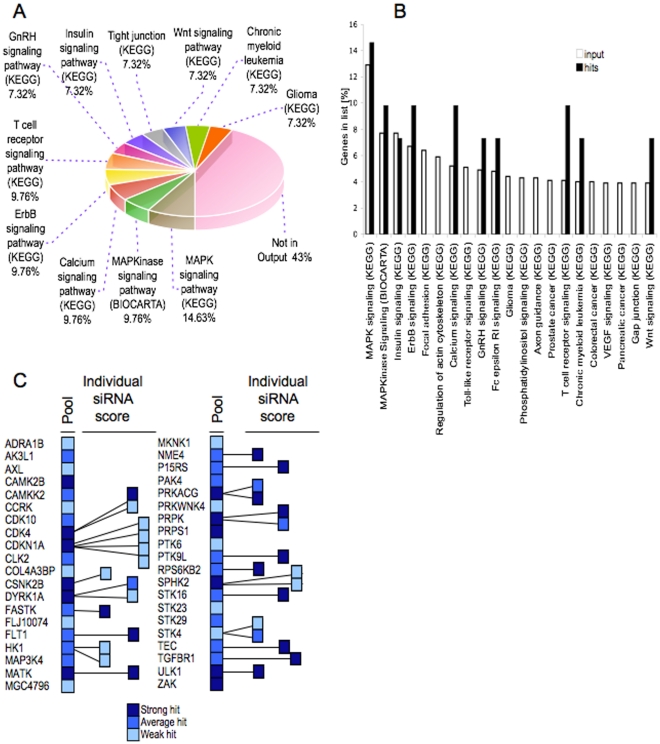
Hit gene-ontology and pathway associations. **A**) **Pathway representation within hit pool.** Hits were analysed for pathway association using the DAVID functional annotation tool (http://david.abcc.ncifcrf.gov/). **B**) **Enrichment for gene ontology.** Pathway association was analysed for hits and input using DAVID. Pathway representation within hits is plotted against that for input targets. **C**) **Hit validation.** Hits were assessed using individual oligonucleotides represented within the pool. The number of active oligonucleotides and level of response is indicated. Hit classification was as for the screen.

To obtain information as to the type of signalling that was detected in the screen we probed for the association of the identified hits with known signalling pathway ontology. To do so we searched for representation of hits within defined pathways and processes using the NIH Database for Annotation, Visualization and Integrated Discovery (DAVID), http://david.abcc.ncifcrf.gov/home.jsp. This revealed considerable representation within MAPK and calcium signalling (Figure 2A) along with membrane receptor signalling ontology in which both MAPK and calcium signalling play a role. Overall, 23 of the 41 hits (57%) were accounted for by these pathway categories. 18 hits (43%) were not represented within the analysis output, indicating that the screen also identified components that do not significantly cluster within the pathways and processes considered in the database interrogated. A number of pathways although strongly represented in the screened gene set were not reflected in the hit list, indicating selectivity of the screen (Figure 2B).

To confirm validity of the hits and ensuing predictions, we sought hit confirmation using individual siRNA duplexes. We confined this analysis to hits that had scored as either strong or average in the primary screen, regardless of whether they were associated within defined pathway ontology or not.

When re-examined in this way, half of the strong hits (6 of 12) and two hits from the weaker category confirmed with two or more oligonucleotides (Figure 2C), together yielding 8 hits validating with multiple oligonucleotides, representing the p53-related protein kinase PRPK/TP53RK, the mammalian sterile 20-like MAPK pathway component serine threonine kinase STK4/MST1, the cyclin dependent kinase CDK4, the dual specificity tyrosine (Y)- phosphorylation-regulated kinase DYRK1A, the glucose-phosphorylating, glycolytic enzyme hexokinase HK1, the cyclic AMP-dependent protein kinase, gamma catalytic subunit PRKACG and p21^CIP1/WAF1^/CDKN1A. Real-time PCR (RT-PCR) analysis (Figure S2) showed that treatment with the respective oligonucleotides led to transcript knockdown in all instances. Corroborating our original analysis, DAVID analysis confirmed representation of MAPK (STK4, and PRKACG) and calcium signalling components (PRKACG) amongst the validated hits, as well as representation of hits that do not group to the annotated pathway ontology (CDK4, DYRK1A, HK1, p21^CIP1/WAF1^, PRPK).

### Effect of target knockdown on IR-mediated p21^CIP1/WAF1^ expression

To explore how the various hits contribute to the radiation response we examined the effects of their knockdown on the IR-induced accumulation of p21^CIP1/WAF1^. As mentioned previously, irradiation of cells induces expression of p21^CIP1/WAF1^ [41], and the cyclin-dependent kinases (CDKs) responsible for phosphorylation of RB1 are inhibited by p21^CIP1/WAF1^ [42], [43], [44] providing a potential mechanism by which IR treatment leads to the accumulation of active RB1 in cells. Our results that siRNA targeting p21^CIP1/WAF1^ leads to radiation-resistant RB1 phosphorylation (Figure 1E an d 2C) supports the critical role of this gene in G_1_ checkpoint activation. We therefore hypothesized that knockdown of at least some of the targets identified act by affecting p21^CIP1/WAF1^ accumulation.

**Figure 3:**
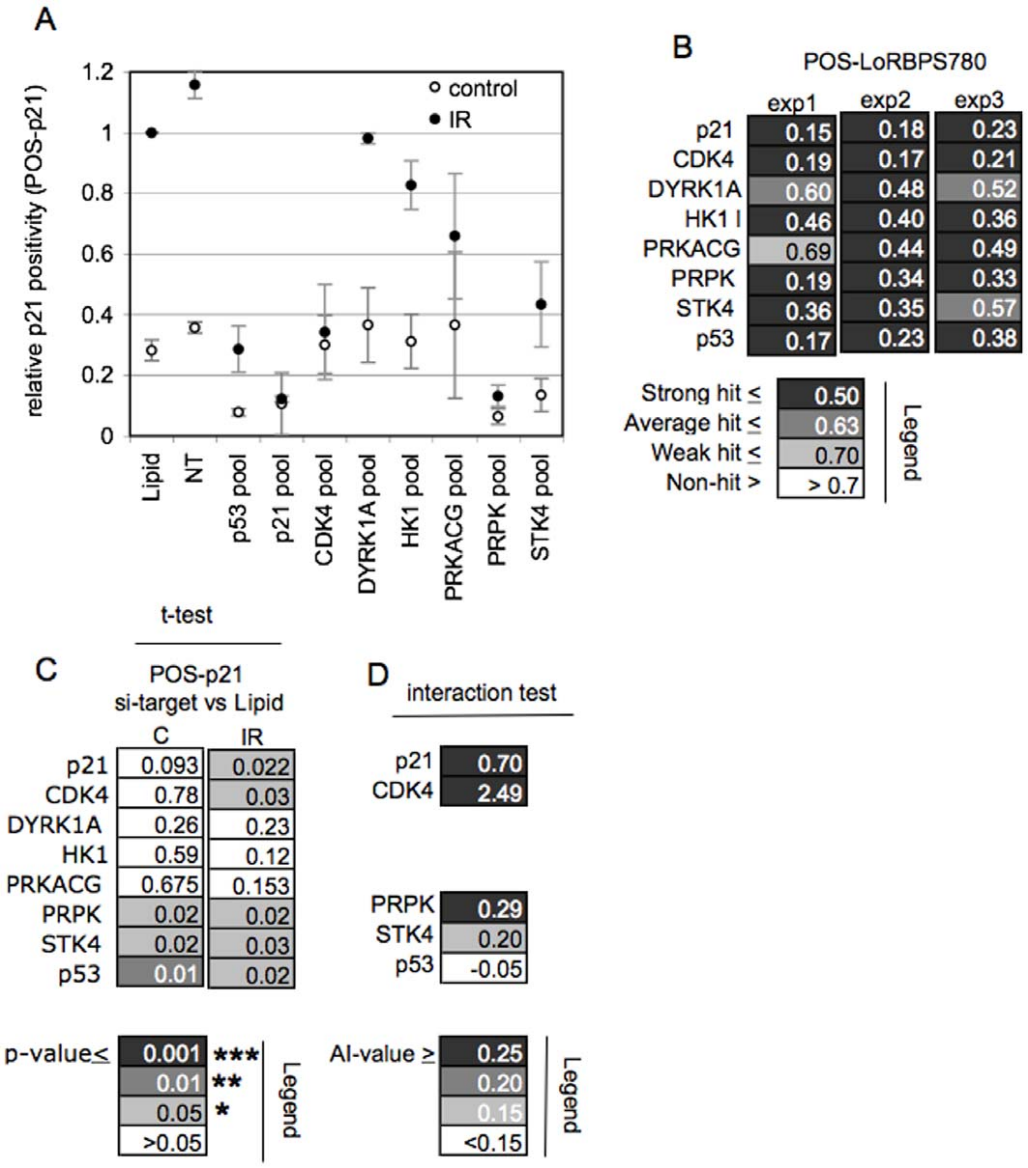
Effect of target knockdown on IR-mediated p21^CIP1/WAF1^ induction. **A**) **Effect of target knockdown on p21^CIP1/WAF1^ positivity.** HCT116 cells transfected with siRNA as indicated were irradiated (IR) or left untreated (control). Cells were assessed for p21^CIP1/WAF1^ positivity 16 hrs post IR. The percentage of cells with p21^CIP1/WAF1^ positivity relative to Mock-treatment (Lipid) is shown. Error bars represent the variance of the mean of three biological replicates, run in triplicate. **B**) **Modulation of RB1 phosphorylation by target knockdown.** POS-LoRBPS780 analysis was performed in parallel plates. Data points represent the means of triplicate technical replicates and are evaluated using hit classification as for the screen. **C**) **Statistical analysis.** Paired t-tests results for data shown in A. **D**) **Treatment interaction test.** Targets that yielded significant impairment of p21^CIP1/WAF1^ positivity were tested for evidence of interaction between radiation and target knockdown. Values indicate the degree of antagonism experienced in IR exposed cells.

To test this hypothesis, we adapted the method for quantifying antibody fluorescence for assessment of p21^CIP1/WAF1^ abundance. To determine the percentage of p21^CIP1/WAF1^-positive cells (POS-p21) we gated for nuclear signal intensity substantially higher than the background fluorescence in cells with ablation of the transcription regulator TP53, known to facilitate p21^CIP1/WAF1^ induction in irradiated cells [45] (Figure S4 and Material and Methods). As expected IR treatment of cells led to a robust increase in the percentage of cells with p21^CIP1/WAF1^ positivity at 16 hrs, the time when RB1 activation is first apparent, in either Mock transfected cells or cells transfected with NT oligonucleotide (Figure 3). A substantial and highly significant reduction in the percentage of p21^CIP1/WAF1^ positive cells was seen upon knockdown of three of the validated targets, PRPK/TP53RK, the MAPK pathway component STK4/MST1 and CDK4 (Figure 3A, C). Notably, knockdown of the remaining three targets, DYRK1A, HK1, and PRKACG, had minor and non-significant effects (Figure 3A, C), although their knockdown effectively prevented IR-induced loss of RB1 phosphorylation in a parallel assessment (Figure 3B).

Knockdown of PRPK and STK4 also reduced p21^CIP1/WAF1^ positivity in the unirradiated cells (Figure 3A, C), indicating the potential involvement of these kinases in signalling contexts independent of IR challenge. Mathematical testing for an interaction between knockdown of these targets and irradiation (see Materials and Methods) provides evidence for a net antagonism of target knockdown with IR-mediated p21^CIP1/WAF1^ accumulation that is not explained by the reduction of p21^CIP1/WAF1^ expression in unchallenged cells (Figure 3D). Hence although PRPK and STK4 promote p21^CIP1/WAF1^ positivity in an IR-independent context, these genes in addition have a significant role in supporting a DNA damage-associated increase in p21^CIP1/WAF1^ positivity.

Together our experiments suggest a significant involvement of a group of the hits in facilitating p21^CIP1/WAF1^ positivity upon irradiation. In contrast other hits must play a role in radiation mediated RB1 activation unconnected to p21^CIP1/WAF1^positivity.

### Effects on IR-mediated G_1_ arrest

**Figure 4:**
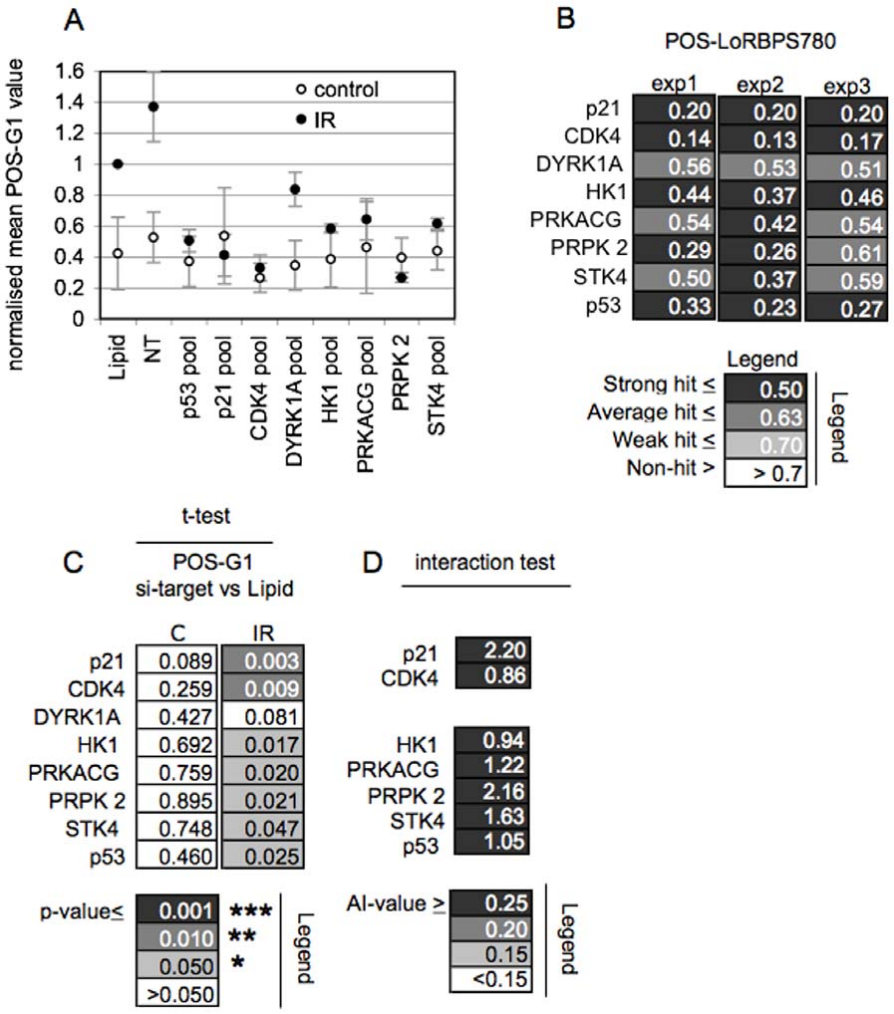
Effect of target knock down on G1 checkpoint activation. **A**) **Effect of target knockdown on relative G1 positivity.** HCT116 cells transfected with siRNA as indicated were irradiated (IR) or left untreated (control). Cells were fixed 16 hours later and assessed for the proportion of cells in G1. The degree of G1 positivity relative to Mock-treated (Lipid) cells is shown. Error bars represent the variance from the mean of three biological replicates, run in triplicate. **B**) **Modulation of RB1 phosphorylation by target knockdown.** POS-LoRBPS780 analysis performed in parallel to A). Data points represent the means of triplicate technical replicates. **C**) **Statistical analysis.** Paired t-tests for data shown in A. **D**) **Treatment interaction test.** Data were assessed for evidence of a interaction between radiation and target knockdown. Values indicate the degree of antagonism experienced in IR exposed cells.

Since DNA damage-induced activation of RB1 is thought to promote cell cycle arrest in G_1_ [29], [46] we tested if the identified hits are required for this response. To assess cell cycle response we used a GFP-tagged cell-cycle reporter that localizes to the nucleus during G1 but redistributes to the cytoplasm as a consequence of CDK2 activation and S phase entry [47]. Using HCT116 cells with stable expression of this reporter we determined the percentage of G_1_ cells following IR exposure and upon knockdown of the various hits (Figure 4). Cells with a ratio of nuclear to cytoplasmic fluorescence of two or greater were considered G_1_ (POS-G1, Figure S4 and Materials and Methods). As previously, we used POS-LoRBPS780 analysis alongside this assessment to monitor for siRNA performance (Figure 4B).

Knockdown of all targets led to loss of G_1_ cells when compared to Mock (Lipid) treatment or treatment with NT oligonucleotide. The cumulative data scored significantly in paired Student's t-tests in all instances except DYRK1A where, however, the calculated p-value (0.08) strongly converged towards significance (Figure 4C). None of the targets when knocked down caused significant changes in the G_1_ content in non-irradiated cells (Figure 4A, C), indicating the encoding genes do not act by affecting normal cell cycle progression. Mathematical testing for interaction between target knockdown and IR confirmed selective antagonism of G_1_ positivity in IR exposed cells as opposed to alteration of G_1_ positivity in unchallenged cells, supporting a significant role and requirement for the identified hits in IR-mediated G_1_ checkpoint activation. Inhibitors of canonical DSB signalling did not prevent the accumulation of cells in G1 following IR exposure, consistent with our earlier results (Figure S1) that such signalling is not involved in RB1 activation, and hence may not participate in the control of G1 checkpoint response (Figure S3).

### Effect of target silencing on IR-associated cell survival

**Figure 5:**
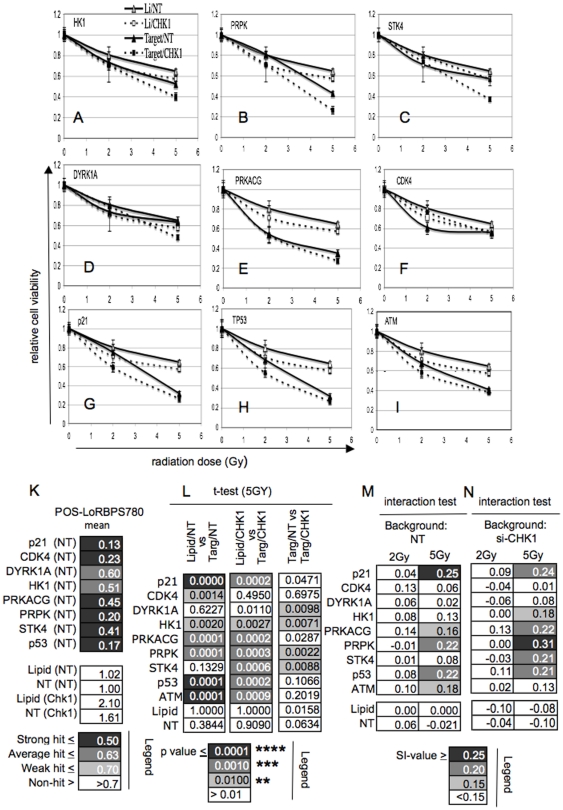
Effect of target knockdown on radiation survival. **A–I**) **Target knockdown affects survival of IR exposed cells.** HCT116 cells transfected with target siRNA alone or in combination with siRNA targeting CHK1. Cells were Mock-irradiated or irradiated with 2 Gy or 5 Gy and viable cells quantified 5 days later. Data plotted are normalized to the respective untreated controls. Filled squares = combined target and CHK1 knockdown (Target/CHK1), open squares = CHK1 only (Li/CHK1), filled triangles = Target only (Target/NT), open triangles = Mock (Li/NT). Error bars represent the variance from the mean from three technical replicates. **K**) **Modulation of RB1 phosphorylation by target knockdown.** Parallel POS-LoRBPS780 analysis was used to verify siRNA performance. **L**) **Statistical analysis.** Student t-test for cell viability data shown in A–I, (Li/NT vs Targ/NT) probing for a significant effect of target knockdown in unperturbed cells, (Li/CHK1 vs Targ/CHK1) probing for a significant effect of target knockdown in CHK1-perturbed cells, Target/NT vs Target/CHK1 probing for a significant effect of CHK1 knockdown in target-perturbed cells. **M, N**) **Treatment interaction.** Assessment for evidence of interaction between radiation and target knockdown. Values represent the degree of net synergism between target knockdown and IR in NT (M) or CHK1-perturbed (N) cell background.

G1 checkpoint activation is thought to play an important role in protecting cells against IR elicited death [6]. Importantly, loss of G1 checkpoint activity was shown to exacerbate the loss of checkpoint functions in S and G2/M phase of the cell cycle, leading to radiation hypersensitivity of cells with such additional defects [48], [49], [50], [51], [52]. We thus hypothesized that ablation of the genes identified in the screen could lead to radio-sensitization and that this may be potentiated by a loss of G2 checkpoint signalling. To test these hypotheses we assessed whether silencing of the identified targets might increase the sensitivity of tumour cells to radiation, using a cell viability assay based on ATP luminescence (Figure 5, Figure S5). To assess if survival loss required (or was exacerbated) by G2 checkpoint loss we simultaneously knocked down the checkpoint kinase CHK1, a key signal transmitter in this checkpoint (Figure 5). As in previous experiments we used parallel-transfected cells for assessment of PS780 RB1 loss, to safe-guard against false negative scores caused by lack of siRNA performance (Figure 5K; Figure S5).

An increased loss of viability, that scored significantly in statistical tests, was seen with all targets. Exacerbation of survival was either confined (STK4 and DYRK1A) or predominant (PRPK and HK1) in cells in which CHK1 expression was knocked down (Figure 5A–D), or equal (PRKACG and CDK4) in both CHK1 perturbed and unperturbed cells (Figure 5E, F and L; Figure S5). Knockdown of STK4, PRPK, HK1 and DYRK1A, which exacerbated survival under condition of CHK1 knockdown, mirrors the effects of RB protein knockdown, where, similarly, enhanced viability loss is dependent upon CHK1 loss (Figure S6), corroborating the prediction that G1 checkpoint control provides radioprotection under conditions of G2 checkpoint loss. We note that combined knockdown of RB1 and RBL1/p107 is required to similarly exacerbate radiation response in these experiments, in agreement with the frequently redundant functioning of these proteins in many cell lineages and signalling contexts [53].

Knockdown of PRKACG or CDK4, where increased loss of viability arises regardless of whether CHK1 function is perturbed (Figure 5L and Figures S5) in turn reflects the effects of p21^CIP1/WAF1^ or TP53 knockdown, or the knockdown of the DNA damage sensor ATM (Figure 5H–I and L). It is known that TP53 and its effector p21^CIP1/WAF1^, as well as ATM, significantly contribute to G_2_/M checkpoint execution [23], [54], [55], explaining why additional CHK1 loss does not exacerbate the loss of viability. The similarity in behaviour of PRKACG and CDK4 may indicate that these targets likewise are required in G2 checkpoint signalling.

We also tested the response to knockdown of the identified targets in TP53-perturbed backgrounds (Figure S7). None of the targets yielded significantly enhanced viability loss here, (Figure S7) although target dependent sensitization was seen in parallel run assays using Mock-perturbed cells, in keeping with a signalling scenario in which TP53 plays a central role.

Mathematical testing for interaction between radiation and target knockdown (Figure 5N; Figure S5) corroborate that target knockdown significantly synergized with IR treatment in reducing cell viability for five of the targets (Figure 5N and Figure S5). Consistent with our prior assessment knockdown of STK4, HK1 and PRPK, which yield enhanced viability loss in conjunction with CHK1 perturbation, yielded substantially enhanced sensitization in CHK1 perturbed cells as opposed to Mock-perturbed or unperturbed cells (Figure 5M, N and Figure S5). Together our experiments provide clear evidence for a modulation of radiation response in cells following perturbation the G1 checkpoint activating targets identified and highlight an interaction with G2 checkpoint competence for the majority of these.

### Data consolidation for derivation of a G_1_ checkpoint-signalling model

**Figure 6:**
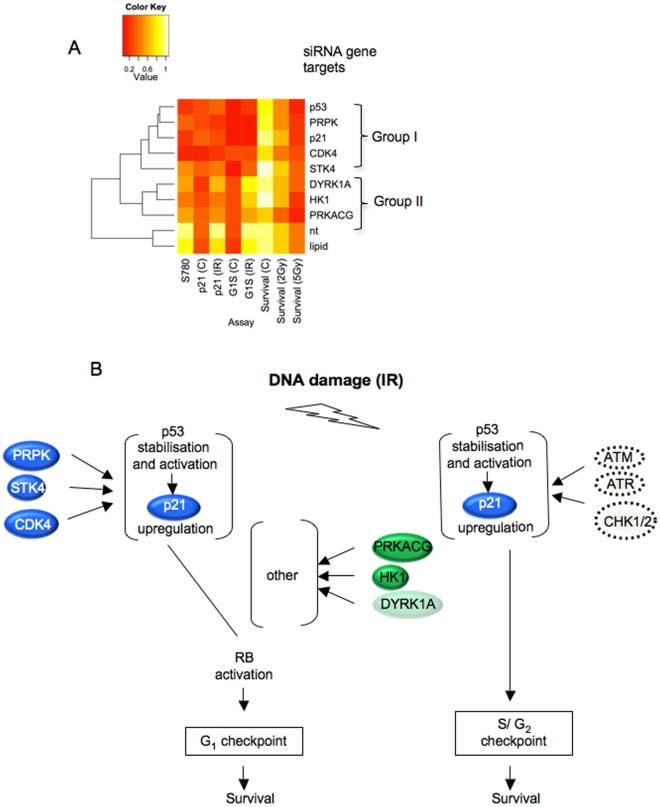
Data consolidation. **A**) **Data clustering analysis.** Unsupervised clustering based on numerical observations. **B**) **Signalling model.** Hits split into groups according to their role in the accumulation of the TP53 target p21^CIP1/WAF1^. Canonical double strand signalling components (ATM, ATR, CHK1/2) affecting the TP53/p21^CIP1/WAF1^ axes in conjunction with S/G2 checkpoint activation do not affect G1 checkpoint function.

To consolidate our data into a signalling model we entered the numerical observations from our analysis into the open access software environment for statistical and graphic data analysis, R (http://www.r-project.org/). Application of the default algorithm for unsupervised clustering analysis sorted the different targets broadly into two groups. One group containing PRPK and STK4, CDK4 and p21^CIP1/WAF1^ (group I) co-clustered with TP53 knockdown, a second group containing DYRK1A, PRKACG and HK1 (group II) aligned separately from the former (Figure 6A). Using the grouping information along with the features established by our experimental analysis, we assembled a signalling framework built around the known axes involving TP53 activation, consequential p21^CIP1/WAF1^ induction and resulting attenuation of RB1 phosphorylation (Figure 6B). According to our experimental results all targets within group I share the feature of being required for p21^CIP1/WAF1^ positivity, predicting that their knockdown either affects transcription of the p21^CIP1/WAF1^ gene, or the subsequent production or accumulation of p21^CIP1/WAF1^ protein. In contrast, group II targets are not required for accumulation of cells with p21^CIP1/WAF1^ positivity, suggesting a network topology whereby p21^CIP1/WAF1^ is required but not sufficient to attenuate RB1 phosphorylation and G1 checkpoint arrest.

## Discussion

Accumulation of RB1 in its active, underphosphorylated form is known to arise in cells experiencing genotoxic stresses, concurrent with arrest of cell cycle progression in such cells [56]. RB1, and in some contexts its paralogues, have been shown to play a critical role in promoting survival of cells exposed to genotoxic stress, supporting a view whereby DNA damage-associated signalling leading to RB1 activation protects cells from radiation-induced death. We used a mechanism-based RNA interference screen to identify kinase signalling required for the accumulation of active RB1 in cells, and identify a group of gene products critically required for RB1 activation and radiation-associated G1 arrest. The majority of these gene products has not previously been linked to IR signalling or G1 checkpoint control.

We found that several of these functions are required for the accumulation of the CDK inhibitor p21^CIP1/WAF1^ in IR-exposed cells. The transcription of p21^CIP1/WAF1^ is activated by TP53, which itself is activated by genotoxic stress [54]. Knockdown of p21^CIP1/WAF1^ or TP53 permits radio-resistant RB1 phosphorylation and prevents G1 arrest, and p21^CIP1/WAF1^ was a hit in the screen, corroborating the critical contribution of this signalling. Two of the screen hits, PRPK and STK4, encode kinases previously linked to TP53 activation and p21^CIP1/WAF1^accumulation, although neither has been linked to checkpoint activation or sensitivity in IR challenged cells. PRPK has been shown to bind to TP53 *in vivo* and along with its yeast homologue Bud32, can phosphorylate mammalian TP53 on S15 and S37 *in vitro* [57]. Phosphorylation of TP53 on these sites is known to result in increased TP53 transcriptional activity [58], supporting an assumption whereby PRPK plays a direct role in regulating signalling through the TP53-p21^CIP1/WAF1^ axis. A very recent report [59] identified PRPK in a screen for sensitizers to spindle assembly checkpoint activation showing that knockdown inhibited mitotic slippage with adaptation, consistent with a role of this gene product in G_1_ checkpoint activation. The second kinase, STK4, is a component of the MAPK ontology, and is not known to directly phosphorylate TP53 [60]. STK4 has been reported to act upstream of the c-Jun N-terminal kinases (JNK1-3/MAPK8-10) and p38/MAPK11-14 mitogen activated kinase family kinases [61], [62]. Both JNK and p38 kinases are known to phosphorylate and activate TP53 [63], [64]. Furthermore p38 family kinases are known to become activated in response to IR and as being required for IR-induced accumulation of p21^CIP1/WAF1^ [65], yet the signalling that leads to their activation is not know. Other recent work showed that STK4 activity promotes p21^CIP1/WAF1^ stability in a JNK-dependent manner [66] suggesting participation of this kinase in signalling resulting in transcriptional as well as posttranscriptional control of p21^CIP1/WAF1^. Our findings, in concert with the current literature, places STK4 as acting through the SAPK/JNK and p38 signalling MAP kinase cascades into DNA damage driven G_1_ checkpoint activation. While it is possible that the p21^CIP1/WAF1^ loss observed in cells with PRPK or STK4 loss is a consequence of checkpoint loss, rather than its cause, the critical requirement of p21^CIP1/WAF1^ accumulation documented by our work together with the known involvement of PRPK and STK4 in the regulation of this CDK inhibitor make such an interpretation less likely.

The canonical route leading to TP53 activation in cells upon genotoxic insult involves ATM or ATR and their substrates CHK1 and CHK2, which in turn facilitate TP53 phosphorylation and activation [67], [68], [69]. As indicated in the results chapter, none of these genes scored in the screen nor did their pharmacological inhibition abolish G1 checkpoint activation, strongly supporting a view whereby signalling implicating these components is not involved in G1 checkpoint control. The implication of the TP53/p21^CIP1/WAF1^ signalling hub in both S/G2 and G1 checkpoint control, along with the documented requirement of PRPK and STK4, suspected to affect this hub, in G1, proposes a model whereby TP53/p21^CIP1/WAF1^ facilitates execution of multiple checkpoints, but executor hub activation is controlled by unrelated yet convergent signalling ontology (see Figure 6B).

STK4 and PRPK cluster with CDK4 as hits through their similar propensity to reduce p21^CIP1/KIP1^ positivity in irradiated cells. Identification of CDK4 in this screen is unexpected, as this kinase is known for its role in promoting RB1 phosphorylation and hence knockdown should lead to attenuation of the event [70]. Knockdown of the closely related and potentially redundant kinase CDK6 did not confer radiation-resistant RB1 phosphorylation but led to loss of RB1 phosphorylation in control and irradiated cells (not shown and Table S1), in line with the perceived role of CDK4/6 in driving RB1 inactivation and indicative of the critical role of this kinase-group in driving RB1 phoshorylation in the cells. It is possible that off-target activities of oligonucleotides led to identification of CDK4 and this cannot be fully excluded, albeit this target validated with two unrelated oligonucleotides. There is no prior published evidence whereby CDK4 is required for the induction or maintenance of p21^Cip1/Kip1^ expression. However, CDK4 in complex with D cyclins can bind p21^Cip1/Kip1^ and it is possible that this interaction stabilizes the CDK inhibitor. Reduction in CDK4 could free cyclin D to activate kinases other than CDK4, capable of phosphorylating RB1, an event that has been seen in cells with CDK4/6 knockout cells [71], and this could explain the radiation-resistant RB1 phosphorylation observed upon CDK4 knockdown.

Several other gene products identified as hits in the screen did not significantly impact p21^CIP1/Waf1^ accumulation, suggesting that they support checkpoint control through mechanisms independent of TP53 activation and p21^Cip1/Kip1^ expression. They include HK1, PRKACG and the DYRK1A dual specificity kinase. There is some evidence that mechanisms other than p21^CIP1/WAF1^-mediated inhibition of the RB1 phosphorylating CDKs may play a role in the DNA damage-associated activation of RB1. For example there is published evidence for the activation of an RB1-directed phosphatase [72] and the phosphorylation-mediated degradation of cyclin D [73], [74], [75] in irradiated cells. It is conceivable that HK1, PRKACG and DYRK1A act through such alternative means.

In common between HK1 and PRKACG is their involvement in driving oxidative glycolysis [76], with knockdown of either enzyme predicted to cause cessation of this process. Identification of HK1 and PRKACG in the screen could hence suggest that glycolytic activity is required for G1 checkpoint activation following genotoxic stress. Short-term treatment of cells with Lonidamine, a selective inhibitor of glycolysis has recently been associated with increased sensitivity to radiation [77], providing corroborating evidence for the interaction of this process with DNA damage response, and potentially checkpoint control.

We note that CDK4, as well as LATS2, a kinase that can activate DYRK1A, was identified in a recently reported RNA interference screen [78] searching for functions which overcome proliferation inhibition following enforced expression of RB1 in RB1 phosphorylation incompetent SAOS2 cells. It is conceivable that knockdown overcame the RB1 phosphorylation incompetence of SAOS2 cells, which is believed to result from high expression of p16INK4A in combination with stabilization of the p21^CIP1/WAF1^ paralogue p27^KIP1^. The functional analysis by Tschoep leaves open whether LATS2 or CDK4 knockdown operates by reinstating RB1 phosphorylation and Tschoep et al. 2011 did not identify other targets that scored positive in the screen reported here.

Radiation affects cell cycle progression at multiple points of the cell cycle. The mechanism underlying DNA damage related G2 and M phase checkpoint control has received considerable attention resulting in increasingly detailed models for the signalling involved [21], [79]. The available knowledge as to signalling involved in damage-associated G1 checkpoint activation is comparatively scarce. Efforts towards delineation of signalling leading to G2/M checkpoint activation may have been fuelled by the recognition that this checkpoint is essential in cells with TP53 loss and thus the potential to uncover therapeutic opportunities in TP53 negative cancers [80]. However, nearly 70% of all cancers express wt TP53, and demonstrate competence for DNA damage-associated TP53 signalling [81]. Inhibition of the components identified here may be a suitable therapeutic strategy to compromise radiation survival of such cancers. The participation of TP53, p21^Cip1/Kip1^ and the RB proteins in G1 checkpoint control is recognized [79]. The identification of druggable kinases required for IR-mediated RB1 activation reported here, to our knowledge, represents the first systematic approach towards discovery of targets for the manipulation of DNA damage-associated G1 checkpoint activation.

## Materials and Methods

### Cell lines and Antibodies

IR was delivered using an AGO HS-MP1 x-ray set. Green fluorescent protein (GFP)-tagged G1 cell cycle reporter encoding the sub-cellular localization domain of human helicase B [47] was purchased from GE Healthcare. CellTiter-Glo® cell viability assay was from Promega. Antibodies used were as follows; anti-RB1-P-S608 (Serotec), anti-RB1-PS795 (Sigma), anti-RB1 (Pharmingen), anti-TP53 (NeoMarkers), anti-p21^CIP1/WAF1^ (Upstate), anti-GAPDH (Abcam), anti-ß-actin (Abcam), Chk1 G-4 sc8408 (Santa Cruz), P-S296 Chk1 2349 S (Cell Signaling), P-S780 Rb 1182-1 (Epitomics). Secondary anti-mouse/rabbit Alexafluor 488/647 (Invitrogen) and secondary HRP-coupled anti-mouse/rabbit IgG (PIERCE). HCT116 human colorectal cancer cells were obtained from ATCC. HCT116 with stable expression of a GFP-tagged cell cycle reporter were generated in house. HCT116 p53+/+ and p53−/− isogenic cell lines were provided by the Vogelstein laboratory. SAR020106 was obtained from Dr. Michelle Garrett, Institute of Cancer Research and used at 1 µM, KU-55933 was obtained form Tocris Bioscience and used at 10 µM.

### Protein analysis

Cell lysates were prepared in HBS buffer (50 mM HEPES pH 7.0, 250 mM NaCl, 0.2% Triton-X100, 1 mM DTT, 1 mM EDTA, 1 mM NaF, 10 mM ß-glycerophosphate, 0.11mM NaVO_4_ plus EDTA free protease inhibitors (Roche). SDS-polyacrylamide gel electrophoresis followed standard procedures. Proteins were transferred onto PVDF membranes (Millipore) then probed with antibodies and HRP-coupled secondary antibodies and exposed to ECL Plus™ reagent (GE Healthcare Sciences). Glutathione-S-transferase (GST)-assisted pull downs used full-length GST-fused E2F-1 with unfused GST as a control. Assays were performed as detailed in [37].

### RNA analysis

RNA was prepared using Trizol (Invitrogen) followed by phenol/chloroform extraction. First strand cDNA synthesis was performed using hexamer random primers (Promega). Quantitative PCR (qPCR) based analysis was performed using the Precision qPCR master-mix (PrimerDesign) with Taqman primers (Applied Biosystems). Water was used instead of cDNA as background control. An Applied Biosystems Prism Sequence Detection System was used to measure relative gene expression from each sample.

### High-throughput siRNA screening assay

HCT116 cells were reverse transfected in triplicate sets of 96-well PackardView plates (Thermofisher) with siRNA from a kinome-covering library (Dharmacon) in a one-gene, one-well format. Cells were seeded at 8,000 cells/well and transfected using HiPerFect lipid transfection reagent (Qiagen) at a fixed siRNA concentration of 20 nM. Cells were exposed to 5 Gy IR 24 hours following transfection and fixed at 48 hrs using 3.7% formaldehyde/PBS (Sigma) for 10 min. Fixed cells were permeabilised using 0.1% Triton X-100 (Sigma) in TRIS-buffered Saline (TBS), blocked in 5% skimmed milk powder in TBS/0.1% Tween-20 (Sigma) and probed with anti-RB1-PS780 rabbit monoclonal antibody (Epitomics) followed by Alexafluor 488 secondary antibody containing 2 mM Hoechst 33258, diluted in 5% skimmed milk powder in TBS/0.1% Tween-20. The intensity of fluorescent nuclear signal in a minimum of 1500 individual cells was determined using an IN Cell Analyzer 3000 high content image analyzer (GE Healthcare). Data were exported into Microsoft Excel, which was used for data handling and analysis. Intensity profiles were established for individual cell data and gated for the percentage of cells displaying low levels of RB1-PS780 loss (POS-LoRBPS780 value – see Figure 4A). All plates contained triplicate positive controls (p21^CIP1/WAF1^ siRNA) and triplicate Mock controls (lipid only). Z-factor calculations relating to these controls were applied to evaluate the quality of all plates. [82]. Plate sets were rejected and rerun where any yielded z-prime <0.2.

### G1 reporter assay and p21^CIP1/WAF1^ positivity

HCT116 cells stably expressing a green fluorescent protein (GFP)-tagged G1 cell cycle reporter were generated by transfection of the reporter alongside a puromycin encoding plasmid (pBabe-Puro). Transfected cultures were subjected to puromycin selection followed by fluorescence activated cell sorting for GFP positivity. siRNA transfection, treatment and fixation were as for screening. Cells were imaged to determine the G1 cell cycle component by determining the nuclear and cytoplasmic GFP signal ratio for a given cell. At a minimum 1500 individual cells were imaged for each data point. The calculated ratio of cytoplasmic: nuclear fluorescence was gated and the percentage of cells per well determined that had a signal intensity ratio >2, (POS-G1 value), see (Figure S4). To quantify p21^CIP1/WAF1^ positivity, cells were stained with anti p21^CIP1/WAF1^ primary antibody, followed by Alexafluor 488 secondary antibody. Nuclear fluorescence for >1500 cells was determined, and cells with objective p21^CIP1/WAF1^ positivity (POS-p21) identified by gating, see (Figure S4)

### Cell survival assay

HCT116 cells were reverse transfected with siRNA and divided three ways into triplicate plates at a seeding density of 2,666 cells/well. A plate at a seeding density of 8000 cells/well for determining POS-LoRBPS780 POS was generated in parallel. 24 hours following transfection plates were irradiated with 5 Gy IR, 2 Gy IR or left untreated. Plates for survival assessment were incubated for a further 5 days. The amount of viable cells per well was assessed using CellTiter-Glo®. Plates for POS-LoRBPS780 assessment were fixed and processed as for the screen. In addition to silencing the various targets we included siRNA duplexes targeting PLK1, a gene previously shown as being required for viability of Ras-transformed cells [83], to provide a positive control for detecting viability loss.

### Statistics

Z-prime calculations were done using 1−(3(σ_p_+σ_n_)/(μ_p_−μ_n_) with _p_ = plate internal positive control or library candidate siRNA, _n_ = plate internal negative controls, σ = standard deviation, μ = mean. All data are expressed as normalized means ± SD from at least three independent experiments unless otherwise stated. Z-scores, describing the distance from the target mean to the population mean in units of the standard error, were calculated using standard Z-test statistics. Gene clustering was performed using the heatmap function in ‘R’ statistical package (http://www.Rproject.org), using the normalized means from three individual experiments for input.

Data from the p21^CIP1/WAF1^ analysis and G1 reporter assays were tested using Student's paired t-test. Tests for interaction between target knockdown and treatment were performed as described [84]. Briefly, the individual effect of target knockdown and treatment was considered. The impact of target knockdown in the absence of IR (Rc) compared to Mock knockdown in the absence of irradiation (Cc) is designated Rc/Cc. The impact of irradiation on Mock-transfected cells is designated C_IR_/C_c_. From these the expected combined response of target knockdown and IR is derived by (Rc/Cc*Cx/Cc). The degree (index) of interaction, either positive (sensitization) or negative (antagonism), is calculated by subtracting the observed combined effect of IR and target knockdown Rx/Cc form the expected interaction, (R_c_/C_c_*C_IR_/C_c_)−(R_IR_/C_c_), where C = Mock-transfected, R = target RNAi tansfected, _IR_ = irradiated, _c_ = untreated. An interaction is considered antagonistic if the effect in C_IR_ exceeds that in R_IR_, and synergistic when the effect in R_IR_ exceeds that in C_IR_.

## Supporting Information

Figure S1 **Modification of RB1 activity by IR.** **A**), **A′**) **Signal quantification for results in** **Figure 1A** **.** Charts depict raw background corrected signal for P-S608 RB1 or relative signal intensity relative to that of pan RB1 in the same samples. Quantification was performed using electronic scans produced from primary autoradiograms. Data were analysed using ImageJ (http://rsbweb.nih.gov/ij/). **B**), **B′**) **Signal quantification for results in** **Figure 1B** **.** Charts depict raw background corrected signal for P-S608 RB1, or P-S608 RB1 signal relative to that of pan RB1 in the same samples. Quantification and analysis was performed as in A. **C**) **IR activates RB1 E2F-binding capacity.** Lysates from IR treated (IR) and control (C) HCT116 cells were incubated with GST-E2F-1 or unfused GST proteins, coupled to Glutathione-Sepharose beads. Material retained on the beads was probed for the presence of RB1 using immunoblotting. Immunoblot analysis of input lysate indicating reduced RB1-PS780 in IR exposed cells. Note increased amount of RB1 signal in pull-down from IR exposed cells. **D**) **Effect of gene knockdown on RB1 phosphorylation in irradiated and control cells.** Z-scores for POS-LoRBPS780 in untreated and irradiated cells.
(TIF)

Figure S2 **Cellular response to target inhibition.** **A**) **IR dependent RB1 activation following pharmacological inhibition of double stand break signalling.** HCT116 cells seeded in 96 well dishes were treated with CHK1 selective inhibitor SAR020106 (1 µM) or the ATM/ATR selective inhibitor KU-55933 (10 µM) for 5 hrs prior to exposure to as indicated. Transfection with siRNA for p53 served as a positive control. NT denotes transfection with NT oligonucleotide, MOCK defers to mock transfected cells. Plates were processed for assessment 24 hrs post IR as for Figure 1E. **B**) **IR dependent CHK1 activation following inhibition of double strand break signalling.** HCT116 seeded in 6 well dishes and treated in parallel to A) were lysed and analysed for CHK1 autophosphorylation activity. **C**) **Signal quantification for results in Figure S2B.** Charts depict background corrected signal for P-S296 CHK1 relative to pan CHK1 in the same samples. Signal detection involved infrared fluorophore-coupled secondary antibodies with signal quantification using a Li-COR Odyssey infrared imager. **D**) **IR dependent RB1 phosphorylation change following pharmacological inhibition of double strand break signalling.** Levels of Ser780 phosphorylated RB1 (RB1-P-S780) and total RB1 (RB1) were established 16 hrs post irradiation by immunoblotting. **E**) **Signal quantification for results in Figure S2D.** Charts depict background corrected signal for P-S780 CHK1 relative to pan RB1 in the same samples. Signal detection and quantification was as for Figure S2C. **F**) **Active siRNA species deplete target mRNA in transfected cells.** HCT116 cells were transfected with single siRNA oligonucleotides as indicated and treated with 5 Gy of IR. RNA was isolated 16 hrs post IR exposure. Transcripts were quantified using Taqman RT/qPCR. Data were normalized against GAPDH. Levels relative to those in cells transfected with NT siRNA are shown. Error bars represent the variance from the mean of triplicate technical replicates. Genes analysed were CDK4, DYRK1A, HK1, SPHK2, STK4, PRPK or p21^CIP1/WAF1^.
(TIF)

Figure S3 **Effect of double stand break signalling inhibition on G1 checkpoint activation.** HCT116 cells seeded in 96 well dishes were treated with CHK1 selective inhibitor SAR020106 (1 µM) or the ATM/ATR selective inhibitor KU-55933 (10 µM) for 5 hrs prior to exposure to IR. Transfection with siRNA for p53 served as a positive control. NT denotes transfection with NT oligonucleotide, MOCK refers to mock transfected cells. Data shown are derived though multiplex analysis of experiments shown in Figure S2A. Data assessment was as for Figure 4A.
(TIF)

Figure S4 **Fixed-cell-assay data evaluation methodology.** **A**) **POS-LoRBS780**, determining the fraction of cells with low RB1-PS780 signal relative to the total number of cells measured. **B**) **POS-p21**, determining the fraction of cells with objective p21^CIP1/WAF1^ positivity relative to the total number of cells measured. **C**) **POS-G1**, determining the fraction of cells with objective G1 positivity relative to the total number of cells measured. Data evaluation relied upon gating for responders based on histogram differences between negative (non-targeting) and positive control (control target), run within the same plate. Example positive (ve+) and negative (ve-) histograms for the different assessments used in the reported work are shown.
(TIF)

Figure S5 **Effect of target knockdown on radiation survival in unperturbed backgrounds.** **A–G**) **Effects of target kockdown on survival of IR exposed cells.** HCT116 cells transfected with target siRNA were irradiated with 2 or 5 Gy, or left untreated (control). Viable cells were quantified 5 days after IR. Data are normalized to the untreated controls. Filled triangles = target (Target), open triangles = Mock (Li). Error bars represent the variance from the mean of three biological replicates, run in triplicate each. **H**) **Modulation of RB1 phosphorylation by target knockdown.** Parallel POS-LoRBPS780 analysis was used to verify siRNA performance. **I**) **Statistical analysis**: Student t-test for data shown in A–G. Note highly significant change in survival for PRKACG (***) and PRPK (**), with HK1 and p21^CIP1/WAF1^ strongly converging towards significance (p<0.05). **K**) **Treatment interaction.** Data were assessed for evidence of interaction between radiation and target knockdown. Values represent the degree of synergism experienced in IR exposed cells.
(TIF)

Figure S6 **Effect of RB knockdown on radiation survival.** **A–E**) **RB family knockdown affects survival of IR exposed cells.** HCT116 cells transfected with oligonucleotides targeting retinoblastoma family proteins either alone, or in combination with siRNA targeting CHK1. siRNA targeting p21^CIP1/WAF1^ and non-targeting (NT) oligonucleotides were run alongside for control. Cells were irradiated with 2 or 5 Gy or left untreated and viable cells were quantified 5 days following IR exposure. Data are normalized to the respective untreated controls. Open triangles = Mock (Li/NT), open squares = CHK1 only (Li/CHK1), filled triangles = target only (target/NT), filled square = combined target and CHK1 knockdown (target/CHK1). Error bars represent the variance from the mean from three technical replicates. **F**) **Modulation of RB1 phosphorylation by target knockdown.** Parallel POS-LoRBPS780 analysis was used to verify siRNA performance. **G**) **Statistical analysis**: Student t-test for data shown in A–I. **H**) **Treatment interaction.** Data were assessed for evidence of interaction between radiation and target knockdown. Values represent the degree of net synergism experienced in IR exposed cells in either Mock-perturbed (NT) or CHK1-perturbed background.
(TIF)

Figure S7 **Interaction of p53 perturbation on survival of cells with target knockdown.** **A–G**) **Effects of target kockdown on survival of IR exposed cells.** HCT116 cells were transfected with target siRNA alone or in combination with siRNA targeting p53. Cells were treated with IR (5 Gy or 2 Gy) or left untreated (control). Viable cells were quantified 5 days after IR. Data are normalized to the untreated controls. Error bars depict the variance from the mean for three technical replicates. Filled square = combined target and p53 knockdown (target/p53) filled triangles = target only (target/NT), open triangles = Mock (Li/NT). **H**) **Modulation of RB1 phosphorylation by target knockdown.** Parallel POS-LoRBPS780 analysis, verifying siRNA performance. **I, K**) **Treatment interaction.** Data were assessed for evidence of interaction between radiation and target knockdown. Values represent the degree of net synergism experienced in IR exposed cells. Note absence of significant synergy in p53-perturbed backgrounds.
(TIF)

Table S1 **Screen data.** Target official gene symbol in alphabetical order; average POS-LoRBPS780 (Average), variation from the mean for n = 3 replicates (Standard Deviat) and Z-score statistics calculated from the average POS-LoRBPS780 (Z-score) are shown for each target.
(PDF)
