# Supplementary figures and images for: Mechanism-Based Screen Establishes Signalling Framework for DNA Damage-Associated G1 Checkpoint Response
Source: PLoS One. 2012 Feb 27;7(2):e31627. doi: 10.1371/journal.pone.0031627 (PMC3288045; doi:10.1371/journal.pone.0031627)

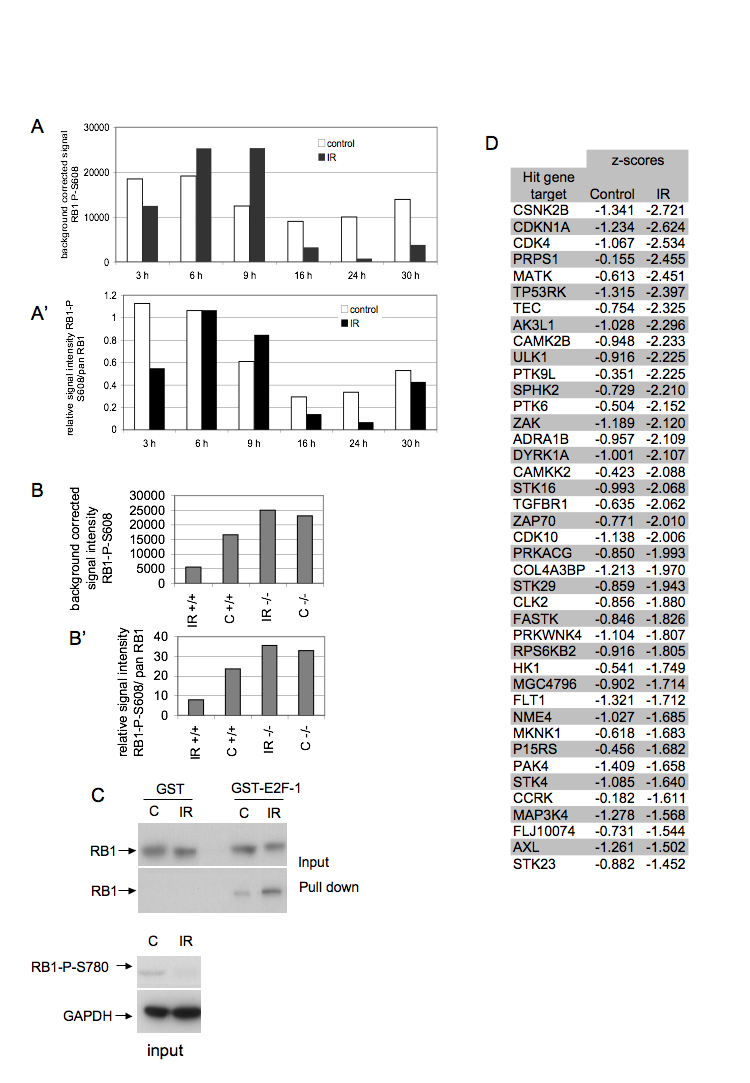

Supplement: Figure S1 — Modification of RB1 activity by IR. A), A′) Signal quantification for results in Figure 1A . Charts depict raw background corrected signal for P-S608 RB1 or relative signal intensity relative to that of pan RB1 in the same samples. Quantification was performed using electronic scans produced from primary autoradiograms. Data were analysed using ImageJ (http://rsbweb.nih.gov/ij/). B), B′) Signal quantification for results in Figure 1B . Charts depict raw background corrected signal for P-S608 RB1, or P-S608 RB1 signal relative to that of pan RB1 in the same samples. Quantification and analysis was performed as in A. C) IR activates RB1 E2F-binding capacity. Lysates from IR treated (IR) and control (C) HCT116 cells were incubated with GST-E2F-1 or unfused GST proteins, coupled to Glutathione-Sepharose beads. Material retained on the beads was probed for the presence of RB1 using immunoblotting. Immunoblot analysis of input lysate indicating reduced RB1-PS780 in IR exposed cells. Note increased amount of RB1 signal in pull-down from IR exposed cells. D) Effect of gene knockdown on RB1 phosphorylation in irradiated and control cells. Z-scores for POS-LoRBPS780 in untreated and irradiated cells. (TIF) [file pone.0031627.s001.tif]

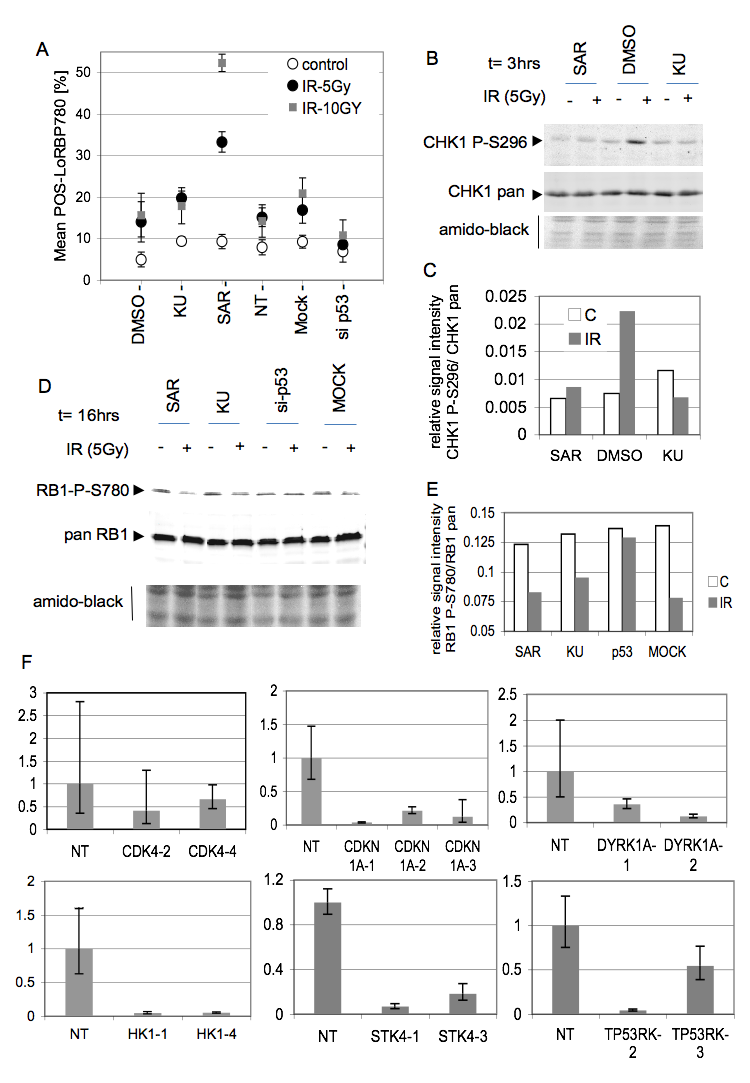

Supplement: Figure S2 — Cellular response to target inhibition. A) IR dependent RB1 activation following pharmacological inhibition of double stand break signalling. HCT116 cells seeded in 96 well dishes were treated with CHK1 selective inhibitor SAR020106 (1 µM) or the ATM/ATR selective inhibitor KU-55933 (10 µM) for 5 hrs prior to exposure to as indicated. Transfection with siRNA for p53 served as a positive control. NT denotes transfection with NT oligonucleotide, MOCK defers to mock transfected cells. Plates were processed for assessment 24 hrs post IR as for Figure 1E. B) IR dependent CHK1 activation following inhibition of double strand break signalling. HCT116 seeded in 6 well dishes and treated in parallel to A) were lysed and analysed for CHK1 autophosphorylation activity. C) Signal quantification for results in Figure S2B. Charts depict background corrected signal for P-S296 CHK1 relative to pan CHK1 in the same samples. Signal detection involved infrared fluorophore-coupled secondary antibodies with signal quantification using a Li-COR Odyssey infrared imager. D) IR dependent RB1 phosphorylation change following pharmacological inhibition of double strand break signalling. Levels of Ser780 phosphorylated RB1 (RB1-P-S780) and total RB1 (RB1) were established 16 hrs post irradiation by immunoblotting. E) Signal quantification for results in Figure S2D. Charts depict background corrected signal for P-S780 CHK1 relative to pan RB1 in the same samples. Signal detection and quantification was as for Figure S2C. F) Active siRNA species deplete target mRNA in transfected cells. HCT116 cells were transfected with single siRNA oligonucleotides as indicated and treated with 5 Gy of IR. RNA was isolated 16 hrs post IR exposure. Transcripts were quantified using Taqman RT/qPCR. Data were normalized against GAPDH. Levels relative to those in cells transfected with NT siRNA are shown. Error bars represent the variance from the mean of triplicate technical replicates. Genes analysed were CDK4, [file pone.0031627.s002.tif]

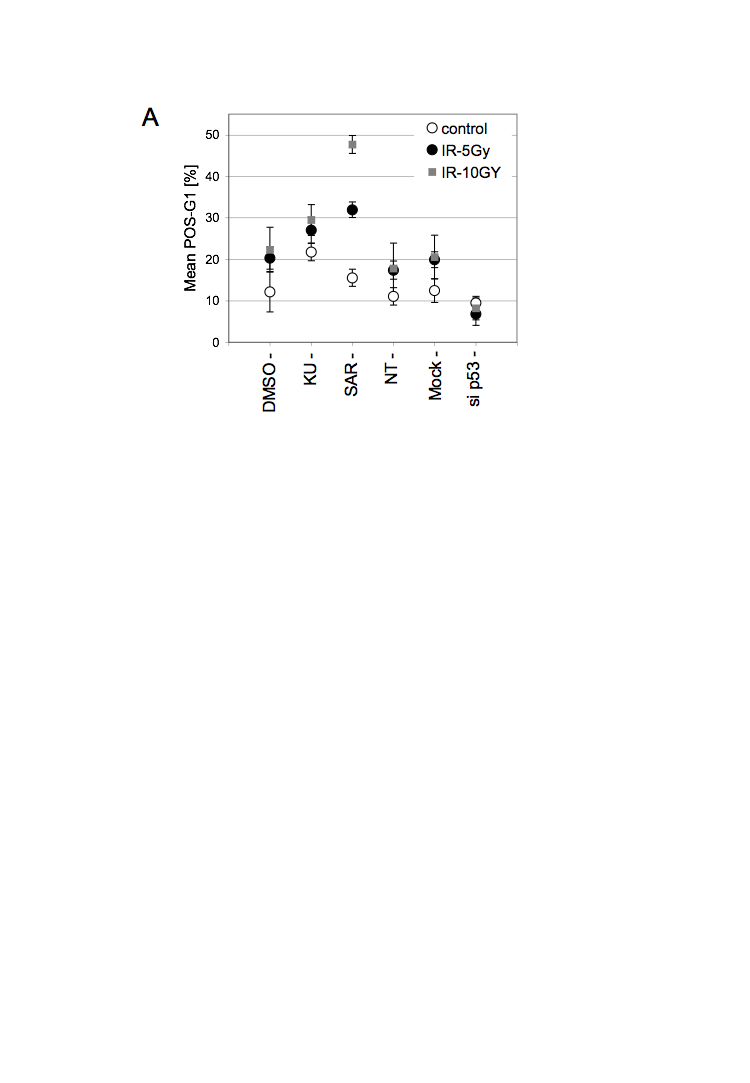

Supplement: Figure S3 — Effect of double stand break signalling inhibition on G1 checkpoint activation. HCT116 cells seeded in 96 well dishes were treated with CHK1 selective inhibitor SAR020106 (1 µM) or the ATM/ATR selective inhibitor KU-55933 (10 µM) for 5 hrs prior to exposure to IR. Transfection with siRNA for p53 served as a positive control. NT denotes transfection with NT oligonucleotide, MOCK refers to mock transfected cells. Data shown are derived though multiplex analysis of experiments shown in Figure S2A. Data assessment was as for Figure 4A. (TIF) [file pone.0031627.s003.tif]

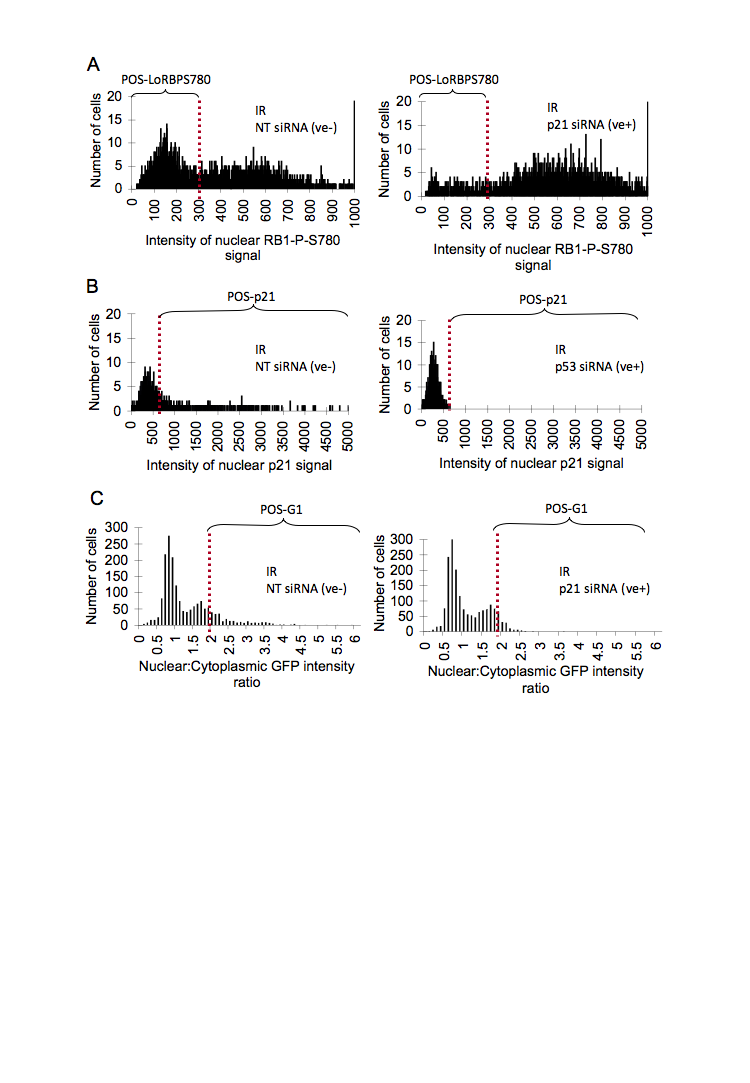

Supplement: Figure S4 — Fixed-cell-assay data evaluation methodology. A) POS-LoRBS780, determining the fraction of cells with low RB1-PS780 signal relative to the total number of cells measured. B) POS-p21, determining the fraction of cells with objective p21CIP1/WAF1 positivity relative to the total number of cells measured. C) POS-G1, determining the fraction of cells with objective G1 positivity relative to the total number of cells measured. Data evaluation relied upon gating for responders based on histogram differences between negative (non-targeting) and positive control (control target), run within the same plate. Example positive (ve+) and negative (ve-) histograms for the different assessments used in the reported work are shown. (TIF) [file pone.0031627.s004.tif]

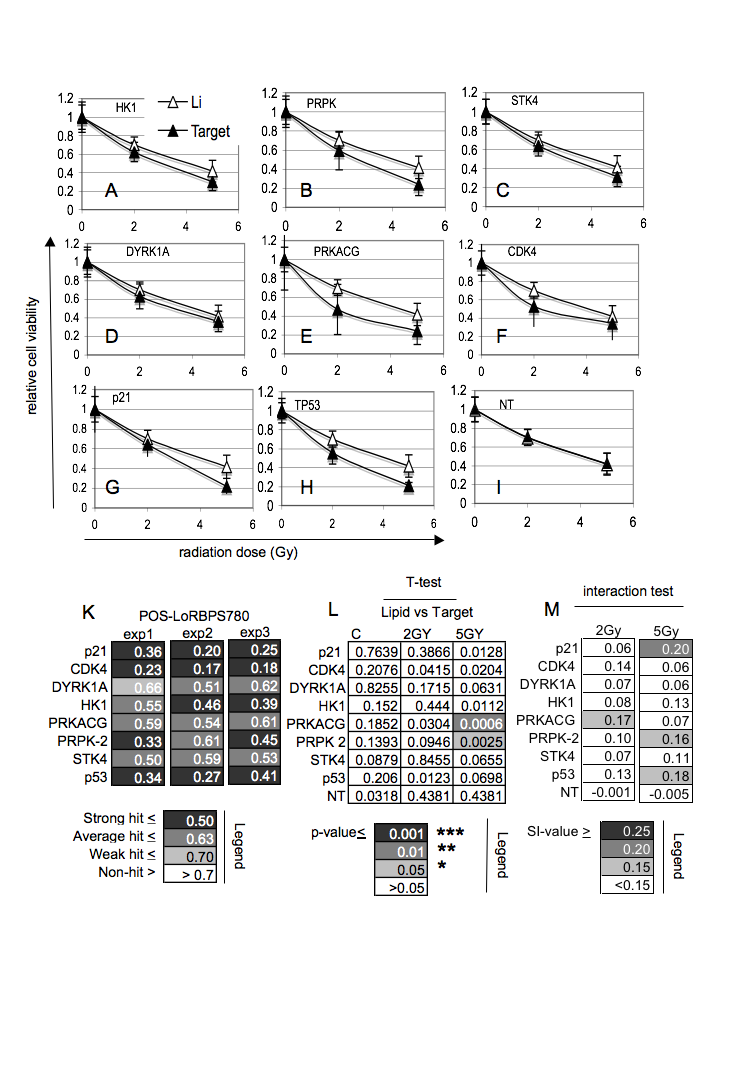

Supplement: Figure S5 — Effect of target knockdown on radiation survival in unperturbed backgrounds. A–G) Effects of target kockdown on survival of IR exposed cells. HCT116 cells transfected with target siRNA were irradiated with 2 or 5 Gy, or left untreated (control). Viable cells were quantified 5 days after IR. Data are normalized to the untreated controls. Filled triangles = target (Target), open triangles = Mock (Li). Error bars represent the variance from the mean of three biological replicates, run in triplicate each. H) Modulation of RB1 phosphorylation by target knockdown. Parallel POS-LoRBPS780 analysis was used to verify siRNA performance. I) Statistical analysis: Student t-test for data shown in A–G. Note highly significant change in survival for PRKACG (***) and PRPK (**), with HK1 and p21CIP1/WAF1 strongly converging towards significance (p<0.05). K) Treatment interaction. Data were assessed for evidence of interaction between radiation and target knockdown. Values represent the degree of synergism experienced in IR exposed cells. (TIF) [file pone.0031627.s005.tif]

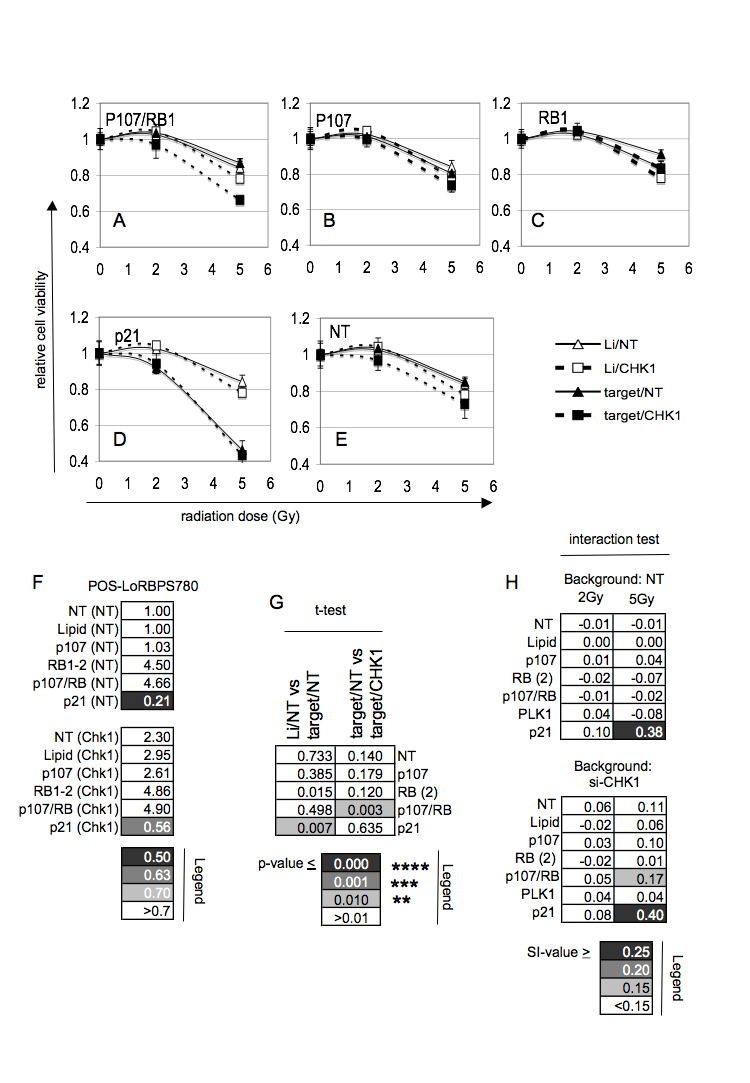

Supplement: Figure S6 — Effect of RB knockdown on radiation survival. A–E) RB family knockdown affects survival of IR exposed cells. HCT116 cells transfected with oligonucleotides targeting retinoblastoma family proteins either alone, or in combination with siRNA targeting CHK1. siRNA targeting p21CIP1/WAF1 and non-targeting (NT) oligonucleotides were run alongside for control. Cells were irradiated with 2 or 5 Gy or left untreated and viable cells were quantified 5 days following IR exposure. Data are normalized to the respective untreated controls. Open triangles = Mock (Li/NT), open squares = CHK1 only (Li/CHK1), filled triangles = target only (target/NT), filled square = combined target and CHK1 knockdown (target/CHK1). Error bars represent the variance from the mean from three technical replicates. F) Modulation of RB1 phosphorylation by target knockdown. Parallel POS-LoRBPS780 analysis was used to verify siRNA performance. G) Statistical analysis: Student t-test for data shown in A–I. H) Treatment interaction. Data were assessed for evidence of interaction between radiation and target knockdown. Values represent the degree of net synergism experienced in IR exposed cells in either Mock-perturbed (NT) or CHK1-perturbed background. (TIF) [file pone.0031627.s006.tif]

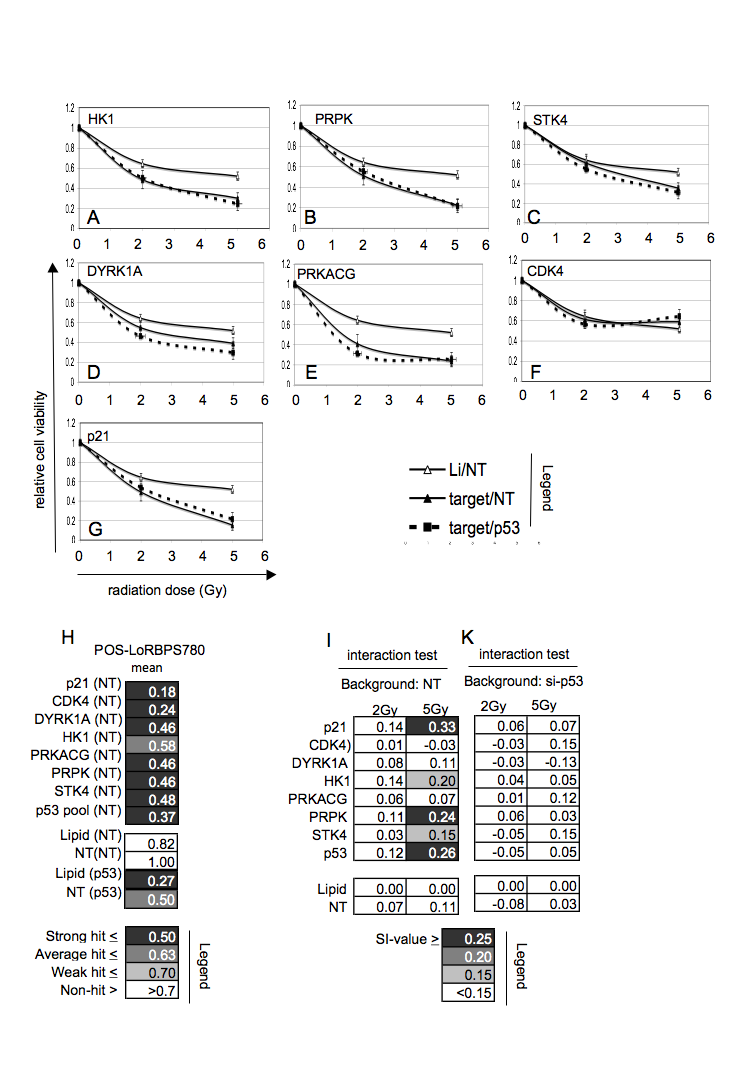

Supplement: Figure S7 — Interaction of p53 perturbation on survival of cells with target knockdown. A–G) Effects of target kockdown on survival of IR exposed cells. HCT116 cells were transfected with target siRNA alone or in combination with siRNA targeting p53. Cells were treated with IR (5 Gy or 2 Gy) or left untreated (control). Viable cells were quantified 5 days after IR. Data are normalized to the untreated controls. Error bars depict the variance from the mean for three technical replicates. Filled square = combined target and p53 knockdown (target/p53) filled triangles = target only (target/NT), open triangles = Mock (Li/NT). H) Modulation of RB1 phosphorylation by target knockdown. Parallel POS-LoRBPS780 analysis, verifying siRNA performance. I, K) Treatment interaction. Data were assessed for evidence of interaction between radiation and target knockdown. Values represent the degree of net synergism experienced in IR exposed cells. Note absence of significant synergy in p53-perturbed backgrounds. (TIF) [file pone.0031627.s007.tif]
