# Supplementary material for: Mechanism-Based Screen Establishes Signalling Framework for DNA Damage-Associated G1 Checkpoint Response
Source: PLoS One. 2012 Feb 27;7(2):e31627. doi: 10.1371/journal.pone.0031627 (PMC3288045; doi:10.1371/journal.pone.0031627)
Supplement: Table S1 — Screen data. Target official gene symbol in alphabetical order; average POS-LoRBPS780 (Average), variation from the mean for n = 3 replicates (Standard Deviat) and Z-score statistics calculated from the average POS-LoRBPS780 (Z-score) are shown for each target. (PDF) [file pone.0031627.s008.pdf]

Hit Definitions

Strong hit	Average POS-LoRBPS780 value < 50 % of the average POS-LoRBPS780 value of negative controls from the plates
Average hit	Average POS-LoRBPS780 value 50 % < 63.5 % of the average POS-LoRBPS780 value of negative controls from the
Poor hit	Average POS-LoRBPS780 value 63.5 % < 70 % of the average POS-LoRBPS780 value of negative controls from the

	siRNA target ge	Hit definition	Hit Accession N	Full Name of Hit	Average	Standard Deviat	Z-score	sorting
395	AAK1	Non-hit	-	-	61.69333333	1.821601859	0.081033589	
42	AATK	Non-hit	-	-	29.70666667	3.227914704	-1.663440759	
185	ABI1	Non-hit	-	-	45.54333333	6.639324765	-0.799747801	
297	ABL1	Non-hit	-	-	54.58666667	2.752314178	-0.306546581	
554	ABL2	Non-hit	-	-	72.05	2.355015924	0.645860788	
354	ACK1	Non-hit	-	-	59.00333333	7.54057248	-0.065672413	
30	ACVR1	Non-hit	-	-	27.77	1.863088833	-1.769061809	
78	ACVR1B	Non-hit	-	-	34.77666667	0.758836829	-1.386935394	
281	ACVR1C	Non-hit	-	-	53.78	1.779410015	-0.350540202	
222	ACVR2	Non-hit	-	-	48.74	1.622251522	-0.625409441	
92	ACVR2B	Non-hit	-	-	35.95333333	2.437546581	-1.32276288	
254	ACVRL1	Non-hit	-	-	51.39	7.44260707	-0.480884941	
57	ADAM9	Non-hit	-	-	32.47	3.407007485	-1.512735336	
101	ADCK1	Non-hit	-	-	36.40666667	4.083997225	-1.298039192	
207	ADCK2	Non-hit	-	-	47.40666667	2.742942459	-0.698126171	
277	ADCK4	Non-hit	-	-	53.46666667	2.900385032	-0.367628634	
300	ADCK5	Non-hit	-	-	54.73	1.664211525	-0.298729532	
365	ADK	Non-hit	-	-	59.67333333	6.724078623	-0.029132257	
335	ADRA1A	Non-hit	-	-	57.49666667	1.368661146	-0.147842318	
15	ADRA1B	Weak hit	NM_000679	adrenergic, alph	21.31	2.73622002	-2.121374365	1
279	ADRB2	Non-hit	-	-	53.69666667	2.462444585	-0.355084998	
93	ADRBK1	Non-hit	-	-	36.00666667	9.929800267	-1.319854211	
155	ADRBK2	Non-hit	-	-	43.12333333	5.545271259	-0.931728665	
520	AGTR2	Non-hit	-	-	70.12	2.385979883	0.540603321	
216	AK1	Non-hit	-	-	48.43666667	5.179057186	-0.641952497	
552	AK2	Non-hit	-	-	71.98	5.173354811	0.642043159	
302	AK3	Non-hit	-	-	54.85666667	0.754077803	-0.291821443	
8	AK3L1	Average hit	NM_016282	adenylate kianse	17.86666667	2.154839515	-2.309165319	2
285	AK5	Non-hit	-	-	53.96333333	3.707861019	-0.340541652	
71	AK7	Non-hit	-	-	34.37666667	3.966942567	-1.408750413	
558	AKAP1	Non-hit	-	-	72.35666667	7.678413465	0.662585635	
566	AKAP11	Non-hit	-	-	73.08	2.427406023	0.702034461	

172	AKAP13	Non-hit	-	-	44.87333333	6.316837289	-0.836287957	
53	AKAP3	Non-hit	-	-	31.66666667	1.755856866	-1.556547166	
48	AKAP4	Non-hit	-	-	30.91	2.78732847	-1.59781391	
80	AKAP5	Non-hit	-	-	34.88	5.421402033	-1.381299847	
244	AKAP6	Non-hit	-	-	50.27666667	6.924805653	-0.54160341	
410	AKAP7	Non-hit	-	-	62.76	4.519435805	0.139206973	
480	AKAP8	Non-hit	-	-	68.23666667	6.879784396	0.43789094	
212	AKT1	Non-hit	-	-	47.94666667	6.093704402	-0.668675895	
123	AKT2	Non-hit	-	-	39.57	5.049940594	-1.12551875	
757	AKT3	Non-hit	-	-	90.57333333	2.760024154	1.656077956	
324	ALK	Non-hit	-	-	56.70333333	9.095242346	-0.191108772	
393	ALS2CR2	Non-hit	-	-	61.57666667	1.833721171	0.074670875	
418	ALS2CR7	Non-hit	-	-	63.14333333	5.358594343	0.160113033	
67	AMHR2	Non-hit	-	-	33.68	2.662649057	-1.446744904	
237	ANGPT4	Non-hit	-	-	49.90666667	1.256436761	-0.561782302	
530	ANKK1	Non-hit	-	-	70.55	3.238101296	0.564054467	
193	ANKRD3	Non-hit	-	-	46.16333333	5.781499229	-0.765934521	
257	APEG1	Non-hit	-	-	51.71333333	5.126639575	-0.463251134	
359	APPL	Non-hit	-	-	59.08666667	1.161737205	-0.061127618	
655	ARAF1	Non-hit	-	-	80.01	1.705021994	1.079979664	
678	ARK5	Non-hit	-	-	81.55333333	11.56362544	1.164149279	
484	ASK	Non-hit	-	-	68.35	4.83325977	0.444071862	
136	ASP	Non-hit	-	-	40.8	1.744505661	-1.058437567	
51	ATM	Non-hit	-	-	31.61666667	1.840036232	-1.559274043	
163	ATR	Non-hit	-	-	43.90666667	6.973107868	-0.889007587	
453	AURKB	Non-hit	-	-	66.13	1.349925924	0.322998507	
334	AURKC	Non-hit	-	-	57.47	4.327805448	-0.149296653	
597	AVPR1A	Non-hit	-	-	75.55666667	4.797085921	0.837105787	
125	AVPR1B	Non-hit	-	-	39.70333333	4.722672266	-1.118247077	
58	AXL	Weak hit	NM_001699	axl receptor tyro	32.48333333	9.982556453	-1.512008169	1
516	AZU1	Non-hit	-	-	69.95666667	1.425143268	0.531695522	
470	BCKDK	Non-hit	-	-	67.27333333	4.096160804	0.385353103	
377	BCR	Non-hit	-	-	60.29666667	3.901029779	0.004862814	
230	BDKRB2	Non-hit	-	-	49.18666667	5.221592988	-0.601049337	
112	BLK	Non-hit	-	-	37.48666667	2.008988137	-1.239138641	
75	BLNK	Non-hit	-	-	34.47666667	6.728613032	-1.403296658	
755	BMP2K	Non-hit	-	-	90.17666667	2.439801905	1.634444729	
379	BMPR1A	Non-hit	-	-	60.44	0.862612312	0.012679863	

127	BMPR1B	Non-hit	-	-	39.74	7.371912913	-1.116247367	
472	BMPR2	Non-hit	-	-	67.46333333	8.711258998	0.395715237	
293	BMX	Non-hit	-	-	54.41666667	6.330721391	-0.315817964	
599	BRAF	Non-hit	-	-	75.68	6.362837417	0.843832084	
337	BRD2	Non-hit	-	-	57.66666667	1.27930971	-0.138570935	
142	BRDT	Non-hit	-	-	41.45333333	5.516677744	-1.022806369	
218	BTK	Non-hit	-	-	48.56333333	6.787711937	-0.635044408	
43	BUB1	Non-hit	-	-	29.87666667	6.164108478	-1.654169376	
466	BUB1B	Non-hit	-	-	67.13	6.517330742	0.377536055	
86	C14ORF20	Non-hit	-	-	35.26333333	5.372991097	-1.360393787	
65	C6ORF199	Non-hit	-	-	33.51333333	3.715929673	-1.455834495	
712	C7ORF16	Non-hit	-	-	84.86666667	4.875144442	1.344850353	
137	C9ORF12	Non-hit	-	-	40.88666667	2.660382178	-1.05371098	
675	C9ORF96	Non-hit	-	-	81.18333333	5.95743513	1.143970387	
214	C9ORF98	Non-hit	-	-	48.32	1.141183596	-0.648315211	
694	CALM3	Non-hit	-	-	82.48333333	5.166046199	1.214869198	
733	CAMK1	Non-hit	-	-	87.11666667	6.983081937	1.467559834	
247	CAMK1D	Non-hit	-	-	50.62333333	7.826968336	-0.52269706	
494	CAMK1G	Non-hit	-	-	68.99333333	6.860060738	0.479157685	
521	CAMK2A	Non-hit	-	-	70.14333333	5.725996274	0.541875864	
9	CAMK2B	Strong hit	NM_001220	calcium/calmodu	19.02333333	3.209615761	-2.246083556	3
594	CAMK2D	Non-hit	-	-	75.13333333	14.93990071	0.814018225	
175	CAMK2G	Non-hit	-	-	45.04666667	8.22655659	-0.826834783	
269	CAMK4	Non-hit	-	-	52.89	9.599473944	-0.39907862	
170	CAMKK1	Non-hit	-	-	44.48666667	6.245929341	-0.857375809	
17	CAMKK2	Average hit	NM_006549	calcium/calmodu	21.70333333	3.745455024	-2.099922929	2
197	CARD10	Non-hit	-	-	46.41	2.096640169	-0.752481926	
68	CARD14	Non-hit	-	-	33.76333333	3.131682189	-1.442200108	
47	CARKL	Non-hit	-	-	30.87666667	6.772594284	-1.599631828	
447	CASK	Non-hit	-	-	65.51	17.55230184	0.289185228	
331	CCL2	Non-hit	-	-	57.19666667	5.430003069	-0.164203582	
74	CCL4	Non-hit	-	-	34.45666667	0.470141823	-1.404387409	
45	CCRK	Weak hit	NM_012119	cell cycle relatec	30.48	3.731849407	-1.621265056	1
642	CD3E	Non-hit	-	-	79.03666667	1.749066418	1.026896452	
211	CD4	Non-hit	-	-	47.90666667	1.026661255	-0.670857397	
306	CD7	Non-hit	-	-	55.01333333	3.991019085	-0.283277227	
429	CDADC1	Non-hit	-	-	64.33666667	7.880116327	0.225194506	
458	CDC2	Non-hit	-	-	66.71333333	7.1702185	0.354812077	

409	CDC2L1	Non-hit	-	-	62.41666667	14.84803129	0.120482415	
340	CDC2L2	Non-hit	-	-	57.74	7.95765669	-0.134571515	
499	CDC2L5	Non-hit	-	-	69.23333333	4.548585861	0.492246696	
631	CDC42BPA	Non-hit	-	-	78.54	5.154192468	0.99980947	
102	CDC42BPB	Non-hit	-	-	36.56666667	8.001301977	-1.289313184	
546	CDC7	Non-hit	-	-	71.77666667	1.427912229	0.630953858	
21	CDK10	Average hit	NM_003674	cyclin dependen	23.20666667	7.239449795	-2.017934817	2
226	CDK11	Non-hit	-	-	48.95666667	9.204223668	-0.613592972	
124	CDK2	Non-hit	-	-	39.60333333	6.495231584	-1.123700832	
565	CDK3	Non-hit	-	-	73.05666667	5.9227049	0.700761919	
3	CDK4	Strong hit	NM_000075	cyclin-dependen	13.48	3.701580743	-2.54840336	3
545	CDK5	Non-hit	-	-	71.76333333	5.104129047	0.630226691	
82	CDK5R1	Non-hit	-	-	34.92	2.541830049	-1.379118345	
190	CDK5R2	Non-hit	-	-	45.98333333	6.864913207	-0.77575128	
25	CDK5RAP1	Non-hit	-	-	24.59333333	5.041639945	-1.942309418	
502	CDK5RAP3	Non-hit	-	-	69.32	7.827956311	0.496973283	
638	CDK6	Non-hit	-	-	78.76333333	4.596012765	1.011989522	
560	CDK7	Non-hit	-	-	72.52	3.58146618	0.671493435	
105	CDK8	Non-hit	-	-	36.80333333	4.313250901	-1.276405965	
318	CDK9	Non-hit	-	-	55.95	3.745810994	-0.232193725	
242	CDKL1	Non-hit	-	-	50.26333333	0.784495592	-0.542330577	
732	CDKL2	Non-hit	-	-	86.80333333	3.440411797	1.450471403	
206	CDKL3	Non-hit	-	-	47.4	4.600576051	-0.698489754	
623	CDKL5	Non-hit	-	-	77.91666667	5.996034801	0.965814399	
438	CDKN1B	Non-hit	-	-	64.93	2.270924922	0.257553451	
63	CDKN1C	Non-hit	-	-	33.33666667	3.609713747	-1.465469462	
113	CDKN2B	Non-hit	-	-	37.56666667	5.850490008	-1.234775637	
278	CDKN2C	Non-hit	-	-	53.68	13.45464975	-0.355993957	
178	CDKN2D	Non-hit	-	-	45.11666667	2.231509205	-0.823017154	
601	CDKN3	Non-hit	-	-	75.82333333	0.563234705	0.851649133	
120	CERK	Non-hit	-	-	39.29666667	8.413289091	-1.14042568	
738	CHEK1	Non-hit	-	-	88.02666667	13.81565175	1.517189002	
456	CHEK2	Non-hit	-	-	66.42333333	7.943578119	0.338996188	
427	CHKA	Non-hit	-	-	63.99666667	2.457485164	0.20665174	
32	CHKB	Non-hit	-	-	28.33333333	1.730154136	-1.738338991	
36	CHRM1	Non-hit	-	-	28.91	2.727874631	-1.706889005	
372	CHUK	Non-hit	-	-	59.99666667	6.49296029	-0.01149845	
355	CINP	Non-hit	-	-	59.02333333	4.43179798	-0.064581662	

708	CIT	Non-hit	-	-	84.30333333	5.26834256	1.314127534	
353	CKB	Non-hit	-	-	58.98666667	5.322371026	-0.066581373	
84	CKM	Non-hit	-	-	35.01666667	5.444100783	-1.373846382	
150	CKMT1	Non-hit	-	-	42.20666667	5.010033267	-0.981721417	
314	CKMT2	Non-hit	-	-	55.47	4.886419957	-0.258371747	
378	CKS1B	Non-hit	-	-	60.37	9.698185397	0.008862235	
233	CKS2	Non-hit	-	-	49.38333333	3.826490995	-0.590323619	
61	CLK1	Non-hit	-	-	33.08	2.305666932	-1.479467432	
26	CLK2	Average hit	NM_001291	cdc-like kinase 2	25.52333333	3.510118706	-1.891589499	2
268	CLK3	Non-hit	-	-	52.88	7.398925598	-0.399623995	
687	CLK4	Non-hit	-	-	81.76666667	3.774775932	1.175783956	
778	CNKSR1	Non-hit	-	-	96.69666667	2.490147251	1.990029538	
291	COASY	Non-hit	-	-	54.16666667	13.27514344	-0.329452351	
23	COL4A3BP	Weak hit	NM_005713	collagen, type IV	23.86333333	4.156228258	-1.982121827	1
274	COPB2	Non-hit	-	-	53.14333333	4.667658228	-0.385262441	
374	CRK7	Non-hit	-	-	60.15333333	2.154653878	-0.002954234	
103	CRKL	Non-hit	-	-	36.6	3.992004509	-1.287495266	
760	CSF1R	Non-hit	-	-	91.21	6.603839792	1.690800195	
600	CSK	Non-hit	-	-	75.78333333	2.874618815	0.849467631	
422	CSNK1A1	Non-hit	-	-	63.48333333	4.332047245	0.178655799	
333	CSNK1A1L	Non-hit	-	-	57.45	4.117268512	-0.150387404	
298	CSNK1D	Non-hit	-	-	54.62666667	2.703041497	-0.304365079	
598	CSNK1E	Non-hit	-	-	75.56666667	3.061002015	0.837651162	
109	CSNK1G1	Non-hit	-	-	37.13	3.871059287	-1.258590366	
81	CSNK1G2	Non-hit	-	-	34.89	3.250169226	-1.380754472	
70	CSNK1G3	Non-hit	-	-	34.29333333	1.28519778	-1.413295208	
69	CSNK2A1	Non-hit	-	-	34.22	6.980408298	-1.417294628	
159	CSNK2A2	Non-hit	-	-	43.52666667	0.652865479	-0.909731855	
1	CSNK2B	Strong hit	NM_001320	casein kinase 2,	10.04333333	1.132445731	-2.735830732	3
183	CXCL10	Non-hit	-	-	45.42333333	1.670159673	-0.806292306	
718	DAPK1	Non-hit	-	-	85.25666667	7.231440613	1.366119996	
596	DAPK2	Non-hit	-	-	75.55666667	5.318311135	0.837105787	
114	DAPK3	Non-hit	-	-	38.26333333	5.365746298	-1.196781145	
626	DCAMKL1	Non-hit	-	-	78.12	12.66217991	0.9769037	
350	DCK	Non-hit	-	-	58.63333333	6.661428776	-0.085851306	
744	DDR1	Non-hit	-	-	88.72333333	11.54634286	1.555183494	
234	DDR2	Non-hit	-	-	49.44	7.192419621	-0.587233158	
719	DGKA	Non-hit	-	-	85.3	4.030334974	1.36848329	

196	DGKB	Non-hit	-	-	46.39333333	0.076376262	-0.753390885	
255	DGKD	Non-hit	-	-	51.55	1.780337047	-0.472158933	
126	DGKE	Non-hit	-	-	39.73666667	10.67899496	-1.116429159	
250	DGKG	Non-hit	-	-	50.96	5.851059733	-0.504336086	
35	DGKI	Non-hit	-	-	28.66333333	1.756170075	-1.7203416	
217	DGKQ	Non-hit	-	-	48.51	5.458213994	-0.637953077	
260	DGKZ	Non-hit	-	-	52.36	3.476046605	-0.42798352	
157	DGUOK	Non-hit	-	-	43.34666667	7.653589572	-0.919548613	
90	DKFZP434C131	Non-hit	-	-	35.68333333	5.1656784	-1.337488018	
713	DKFZp434C141	Non-hit	-	-	84.86666667	4.443437108	1.344850353	
703	DKFZP586B162	Non-hit	-	-	83.39666667	6.426230103	1.264680158	
769	DKFZP761P042	Non-hit	-	-	93.32	2.23577727	1.80587442	
460	DLG1	Non-hit	-	-	66.80666667	2.663763002	0.359902248	
504	DLG2	Non-hit	-	-	69.39333333	1.221733741	0.500972704	
584	DLG3	Non-hit	-	-	74.32666667	7.062310764	0.770024604	
669	DLG4	Non-hit	-	-	80.82666667	9.618951779	1.124518661	
625	DMPK	Non-hit	-	-	78.10333333	9.926980071	0.975994741	
219	DNAJC3	Non-hit	-	-	48.62333333	7.211104862	-0.631772155	
750	DOK1	Non-hit	-	-	89.72666667	5.267222545	1.609902833	
282	DTYMK	Non-hit	-	-	53.84	1.585465231	-0.34726795	
698	DUSP1	Non-hit	-	-	82.89	7.382282845	1.237047801	
621	DUSP10	Non-hit	-	-	77.44666667	7.265984677	0.940181751	
119	DUSP2	Non-hit	-	-	38.97666667	1.808544535	-1.157877695	
754	DUSP22	Non-hit	-	-	90.15333333	2.778710732	1.633172186	
204	DUSP4	Non-hit	-	-	47.33666667	18.03732889	-0.701943799	
563	DUSP5	Non-hit	-	-	72.75	15.21829163	0.684037071	
143	DUSP6	Non-hit	-	-	41.49	4.275078947	-1.020806659	
615	DUSP7	Non-hit	-	-	77.03	2.667358244	0.917457773	
553	DUSP8	Non-hit	-	-	72.03666667	1.110060058	0.64513362	
16	DYRK1A	Strong hit	NM_001396	dual-specificity t	21.35	2.305666932	-2.119192863	3
613	DYRK1B	Non-hit	-	-	76.80333333	8.23192768	0.905095929	
413	DYRK2	Non-hit	-	-	62.86666667	7.683497468	0.145024311	
426	DYRK3	Non-hit	-	-	63.85333333	4.070827107	0.198834691	
158	DYRK4	Non-hit	-	-	43.46	5.810516328	-0.913367691	
177	EDN2	Non-hit	-	-	45.11333333	5.598967167	-0.823198946	
536	EEF2K	Non-hit	-	-	71.01	2.599769221	0.589141738	
448	EGFR	Non-hit	-	-	65.82666667	6.173980348	0.306455451	
514	EIF2AK3	Non-hit	-	-	69.91666667	3.58246191	0.52951402	

603	EIF2AK4	Non-hit	-	-	76.08666667	4.726323448	0.866010687	
683	EKI1	Non-hit	-	-	81.67333333	2.10272046	1.170693785	
485	EPHA1	Non-hit	-	-	68.46333333	9.425626416	0.450252784	
162	EPHA2	Non-hit	-	-	43.88666667	4.430534204	-0.890098337	
729	EPHA3	Non-hit	-	-	86.51333333	2.244422717	1.434655514	
593	EPHA4	Non-hit	-	-	74.92	5.686009145	0.802383549	
346	EPHA5	Non-hit	-	-	58.16333333	4.989652627	-0.111483953	
590	EPHA7	Non-hit	-	-	74.84	6.423363605	0.798020545	
444	EPHA8	Non-hit	-	-	65.42666667	3.329164059	0.284640432	
271	EPHB1	Non-hit	-	-	52.96333333	12.33815356	-0.395079199	
440	EPHB2	Non-hit	-	-	65.03666667	5.191746655	0.263370789	
657	EPHB3	Non-hit	-	-	80.03	5.703709319	1.081070415	
181	EPHB4	Non-hit	-	-	45.22	6.385992484	-0.817381608	
685	EPHB6	Non-hit	-	-	81.74666667	10.40986231	1.174693205	
203	ERBB2	Non-hit	-	-	47.12	12.96627549	-0.713760268	
111	ERBB3	Non-hit	-	-	37.45333333	11.61240859	-1.240956559	
664	ERBB4	Non-hit	-	-	80.35	4.014523633	1.098522431	
145	ERK8	Non-hit	-	-	41.82666667	14.66146082	-1.002445685	
772	ERN1	Non-hit	-	-	94.17	3.383415434	1.852231335	
666	EVI1	Non-hit	-	-	80.52666667	5.533157628	1.108157397	
777	EXOSC10	Non-hit	-	-	96.08	2.073571798	1.95639805	
27	FASTK	Average hit	NM_006712	fas-activated ser	26.53	2.740273709	-1.836688368	2
537	FER	Non-hit	-	-	71.02	7.678404782	0.589687114	
469	FES	Non-hit	-	-	67.18666667	11.61817685	0.380626516	
727	FGFR1	Non-hit	-	-	86.22333333	0.04163332	1.418839625	
762	FGFR2	Non-hit	-	-	91.46333333	1.404789427	1.704616373	
439	FGFR3	Non-hit	-	-	64.97	8.538155539	0.259734952	
330	FGFR4	Non-hit	-	-	57.18333333	6.559186941	-0.16493075	
482	FGR	Non-hit	-	-	68.30333333	14.14756634	0.441526777	
54	FLJ10074	Weak hit	NM_017988	scy1-like 2 (s.ce	31.70666667	8.086410411	-1.554365664	1
766	FLJ10761	Non-hit	-	-	92.15333333	3.021726879	1.742247281	
686	FLJ10842	Non-hit	-	-	81.75666667	0.757451869	1.175238581	
140	FLJ12476	Non-hit	-	-	41.34666667	3.118739061	-1.028623708	
133	FLJ13052	Non-hit	-	-	40.32	5.972712951	-1.08461559	
619	FLJ20574	Non-hit	-	-	77.38333333	0.933880792	0.936727707	
424	FLJ23074	Non-hit	-	-	63.76333333	3.22909172	0.193926312	
706	FLJ23356	Non-hit	-	-	83.64333333	5.252735795	1.278132753	
323	FLJ25006	Non-hit	-	-	56.66	14.1478585	-0.193472066	

187	FLJ32685	Non-hit	-	-	45.70333333	9.181646548	-0.791021793	
534	FLJ34389	Non-hit	-	-	70.84666667	2.893049141	0.580233939	
200	FLJ35107	Non-hit	-	-	46.51333333	7.752472724	-0.74684638	
34	FLT1	Average hit	NM_002019	fms-related tyros	28.63	2.899948275	-1.722159518	2
548	FLT3	Non-hit	-	-	71.81666667	9.946237144	0.63313536	
539	FLT4	Non-hit	-	-	71.43666667	4.250474483	0.612411092	
332	FN3K	Non-hit	-	-	57.36666667	5.505309558	-0.154932199	
634	FN3KRP	Non-hit	-	-	78.58333333	8.291986091	1.002172764	
627	FRAP1	Non-hit	-	-	78.16333333	1.982985964	0.979266994	
104	FRDA	Non-hit	-	-	36.71666667	4.955414547	-1.281132552	
538	FRK	Non-hit	-	-	71.39333333	2.971890532	0.610047798	
746	FUK	Non-hit	-	-	88.98333333	1.121888289	1.569363256	
473	FYB	Non-hit	-	-	67.50666667	4.968121711	0.398078531	
312	FYN	Non-hit	-	-	55.3	4.561260791	-0.26764313	
348	GAK	Non-hit	-	-	58.42666667	2.922470416	-0.097122399	
425	GALK1	Non-hit	-	-	63.84666667	1.553200996	0.198471108	
691	GALK2	Non-hit	-	-	82.20666667	5.541455886	1.199780477	
195	GAP43	Non-hit	-	-	46.26333333	2.927905964	-0.760480767	
692	GCK	Non-hit	-	-	82.36666667	5.271549424	1.208506484	
496	GFRA2	Non-hit	-	-	69.14	6.688370504	0.487156525	
624	GK	Non-hit	-	-	77.94	4.685520249	0.967086941	
295	GK2	Non-hit	-	-	54.45333333	1.371471229	-0.313818254	
91	GMFB	Non-hit	-	-	35.77	6.609364569	-1.33276143	
421	GMFG	Non-hit	-	-	63.43	6.078001316	0.175747129	
97	GNE	Non-hit	-	-	36.23	0.547448628	-1.307674158	
225	GRK1	Non-hit	-	-	48.94666667	9.432477582	-0.614138348	
420	GRK4	Non-hit	-	-	63.36666667	23.25450781	0.172293085	
667	GRK5	Non-hit	-	-	80.60666667	12.34743023	1.112520401	
436	GRK6	Non-hit	-	-	64.86333333	13.30005388	0.253917614	
309	GRK7	Non-hit	-	-	55.14333333	14.70985157	-0.276187346	
384	GSG2	Non-hit	-	-	60.99	4.38620565	0.042675514	
416	GSK3A	Non-hit	-	-	63.04333333	7.365000566	0.154659278	
326	GSK3B	Non-hit	-	-	56.81	15.01155555	-0.185291434	
695	GTF2H1	Non-hit	-	-	82.52	2.277191252	1.216868908	
287	GUCY2C	Non-hit	-	-	54.00666667	3.492425136	-0.338178358	
512	GUCY2D	Non-hit	-	-	69.84333333	3.243552579	0.5255146	
236	GUCY2F	Non-hit	-	-	49.47666667	5.940726667	-0.585233448	
338	GUK1	Non-hit	-	-	57.72666667	2.097744821	-0.135298682	

490	HAK	Non-hit	-	-	68.61666667	2.053030281	0.458615208	
488	HCK	Non-hit	-	-	68.52333333	3.40125467	0.453525037	
665	HIPK1	Non-hit	-	-	80.48333333	6.400510396	1.105794104	
329	HIPK2	Non-hit	-	-	57.07333333	9.206054167	-0.17092988	
296	HIPK3	Non-hit	-	-	54.53666667	5.052091976	-0.309273458	
316	HIPK4	Non-hit	-	-	55.82666667	9.040665536	-0.238920022	
31	HK1	Average hit	NM_000188	hexokinase 1	27.94333333	4.208138939	-1.759608634	2
756	HK2	Non-hit	-	-	90.21	3.677675897	1.636262647	
215	HK3	Non-hit	-	-	48.38333333	7.462294107	-0.644861166	
328	HRI	Non-hit	-	-	57.01	3.761396017	-0.174383924	
585	HSMDPKIN	Non-hit	-	-	74.39	2.514179787	0.773478648	
684	HSPB8	Non-hit	-	-	81.69	0.989292677	1.171602744	
462	HUNK	Non-hit	-	-	66.88	16.91587716	0.363901668	
524	ICK	Non-hit	-	-	70.35333333	8.221443507	0.553328749	
441	IGF1R	Non-hit	-	-	65.04	11.2596936	0.263552581	
153	IHPK1	Non-hit	-	-	42.98333333	2.17488697	-0.939363922	
527	IHPK2	Non-hit	-	-	70.46	6.083970743	0.559146087	
259	IHPK3	Non-hit	-	-	52	7.960709767	-0.447617037	
273	IKBKAP	Non-hit	-	-	53.07666667	4.035298419	-0.388898277	
740	IKBKB	Non-hit	-	-	88.23	4.711273713	1.528278304	
707	IKBKE	Non-hit	-	-	84.03	2.171451128	1.299220605	
152	IL2	Non-hit	-	-	42.64	2.23702928	-0.95808848	
305	ILK	Non-hit	-	-	54.98333333	3.946470997	-0.284913354	
526	ILKAP	Non-hit	-	-	70.40333333	5.999344409	0.556055626	
681	IMPK	Non-hit	-	-	81.59666667	5.200157049	1.166512573	
491	INSR	Non-hit	-	-	68.78	8.37668789	0.467523008	
286	INSRR	Non-hit	-	-	53.97	1.136485812	-0.340178068	
680	IRAK1	Non-hit	-	-	81.57666667	1.392743097	1.165421822	
213	IRAK2	Non-hit	-	-	47.96333333	4.062331022	-0.667766936	
773	IRAK3	Non-hit	-	-	94.86	1.692306119	1.889862243	
716	IRS1	Non-hit	-	-	84.91666667	9.80615283	1.34757723	
562	ITGB1BP1	Non-hit	-	-	72.62	6.79103085	0.67694719	
266	ITK	Non-hit	-	-	52.77333333	5.511500098	-0.405441333	
730	ITPK1	Non-hit	-	-	86.54333333	3.833410144	1.43629164	
221	ITPKA	Non-hit	-	-	48.64333333	5.124532499	-0.630681404	
571	ITPKB	Non-hit	-	-	73.4	6.988476229	0.719486477	
294	ITPKC	Non-hit	-	-	54.41666667	1.470011338	-0.315817964	
602	JAK1	Non-hit	-	-	75.99	6.734144341	0.860738724	

327	JAK2	Non-hit	-	-	56.86	9.839557917	-0.182564557
471	JAK3	Non-hit	-	-	67.38	2.705790088	0.391170442
564	JIK	Non-hit	-	-	72.84333333	6.11681562	0.689127242
580	KDR	Non-hit	-	-	74.00333333	8.013827633	0.752390797
679	KHK	Non-hit	-	-	81.55333333	1.03122904	1.164149279
525	KIAA0999	Non-hit	-	-	70.36	14.59894174	0.553692333
477	KIAA1361	Non-hit	-	-	67.83333333	9.681706117	0.41589413
569	KIAA1399	Non-hit	-	-	73.33666667	1.308485129	0.716032432
468	KIAA1639	Non-hit	-	-	67.18	3.814288925	0.380262932
342	KIAA1765	Non-hit	-	-	57.92333333	4.948093909	-0.124572965
325	KIAA1804	Non-hit	-	-	56.78666667	15.91499084	-0.186563977
551	KIAA1811	Non-hit	-	-	71.86333333	4.799295087	0.635680445
385	KIF13B	Non-hit	-	-	61.14333333	5.924604066	0.051037938
616	KIS	Non-hit	-	-	77.07	2.227307792	0.919639275
498	KIT	Non-hit	-	-	69.16666667	4.021396938	0.488610859
579	KSR2	Non-hit	-	-	73.94333333	2.553435594	0.749118544
360	LAK	Non-hit	-	-	59.11	3.06621917	-0.059855075
366	LCK	Non-hit	-	-	59.73666667	5.541681454	-0.025678212
258	LCP2	Non-hit	-	-	51.86666667	2.272010857	-0.45488871
149	LIM	Non-hit	-	-	42.2	1.74502149	-0.982085001
423	LIMK1	Non-hit	-	-	63.64666667	7.100347409	0.187563598
432	LIMK2	Non-hit	-	-	64.74666667	2.07271159	0.2475549
55	LMTK2	Non-hit	-	-	31.93333333	1.233463957	-1.54200382
725	LMTK3	Non-hit	-	-	86.17	4.896233246	1.415930956
547	LOC115704	Non-hit	-	-	71.78333333	3.843986646	0.631317442
387	LOC149420	Non-hit	-	-	61.20666667	9.180099854	0.054491983
368	LOC340371	Non-hit	-	-	59.89	6.474357111	-0.017315788
617	LOC91807	Non-hit	-	-	77.15	6.077260896	0.924002279
403	LRRK1	Non-hit	-	-	61.95333333	9.836881281	0.095213351
100	LTK	Non-hit	-	-	36.38333333	2.410608499	-1.299311734
690	LYK5	Non-hit	-	-	81.96	9.08922989	1.186327882
129	LYN	Non-hit	-	-	39.98333333	1.859740125	-1.102976564
743	MAGI-3	Non-hit	-	-	88.38666667	1.748723344	1.536822519
272	MAK	Non-hit	-	-	52.99666667	9.173943173	-0.393261281
628	MALT1	Non-hit	-	-	78.31666667	0.815250473	0.987629418
134	MAP2K1	Non-hit	-	-	40.42	2.514338879	-1.079161835
500	MAP2K1IP1	Non-hit	-	-	69.26	1.89649677	0.493701031
166	MAP2K2	Non-hit	-	-	44.10333333	1.295080435	-0.878281869

689	MAP2K3	Non-hit	-	-	81.85	7.649633978	1.180328752	
246	MAP2K4	Non-hit	-	-	50.50666667	7.611841652	-0.529059774	
223	MAP2K5	Non-hit	-	-	48.90333333	3.962440326	-0.616501642	
349	MAP2K6	Non-hit	-	-	58.43	14.90321106	-0.096940607	
249	MAP2K7	Non-hit	-	-	50.84333333	3.717019415	-0.5106988	
180	MAP3K1	Non-hit	-	-	45.20333333	7.380246157	-0.818290567	
653	MAP3K10	Non-hit	-	-	79.69666667	4.168241036	1.062891233	
508	MAP3K11	Non-hit	-	-	69.65333333	3.891353663	0.515152466	
405	MAP3K12	Non-hit	-	-	62.25	9.02487119	0.111392824	
483	MAP3K13	Non-hit	-	-	68.34666667	4.952901507	0.443890071	
171	MAP3K14	Non-hit	-	-	44.49	10.11468734	-0.857194017	
535	MAP3K2	Non-hit	-	-	70.90666667	3.188798102	0.583506192	
542	MAP3K3	Non-hit	-	-	71.51666667	8.55760091	0.616774096	
50	MAP3K4	Average hit	NM_005922	mitogen-activate	31.27	10.01025474	-1.578180393	2
641	MAP3K5	Non-hit	-	-	79.00333333	3.964357872	1.025078533	
364	MAP3K6	Non-hit	-	-	59.45	9.483422378	-0.041312309	
415	MAP3K7	Non-hit	-	-	63.02333333	11.83096502	0.153568527	
568	MAP3K8	Non-hit	-	-	73.26333333	5.323404299	0.712033012	
736	MAP3K9	Non-hit	-	-	87.62333333	4.238399855	1.495192192	
759	MAP4K1	Non-hit	-	-	90.93666667	3.83970485	1.675893265	
98	MAP4K2	Non-hit	-	-	36.25666667	6.016122782	-1.306219824	
288	MAP4K3	Non-hit	-	-	54.13	15.67140389	-0.331452061	
313	MAP4K4	Non-hit	-	-	55.46666667	0.771513664	-0.258553539	
238	MAP4K5	Non-hit	-	-	49.93	11.4477203	-0.56050976	
702	MAPK1	Non-hit	-	-	83.39333333	9.508313906	1.264498366	
645	MAPK10	Non-hit	-	-	79.11666667	4.104099576	1.031259456	
629	MAPK11	Non-hit	-	-	78.41333333	9.648659665	0.992901381	
735	MAPK12	Non-hit	-	-	87.56333333	5.850002849	1.491919939	
734	MAPK13	Non-hit	-	-	87.36666667	7.76264345	1.481194221	
256	MAPK14	Non-hit	-	-	51.7	9.647709573	-0.463978301	
578	MAPK3	Non-hit	-	-	73.72333333	3.827497006	0.737120284	
644	MAPK4	Non-hit	-	-	79.06666667	1.597164154	1.028532578	
465	MAPK6	Non-hit	-	-	67.05666667	2.399527731	0.373536635	
714	MAPK7	Non-hit	-	-	84.87333333	7.728417259	1.345213936	
205	MAPK8	Non-hit	-	-	47.37333333	4.128684213	-0.699944089	
724	MAPK8IP1	Non-hit	-	-	86.11666667	6.507759471	1.413022287	
722	MAPK8IP2	Non-hit	-	-	85.94333333	5.520184176	1.403569112	
611	MAPK8IP3	Non-hit	-	-	76.42666667	7.124193522	0.884553453	

356	MAPK9	Non-hit	-	-	59.05666667	3.911806914	-0.062763744	
62	MAPKAPK2	Non-hit	-	-	33.31666667	19.89993551	-1.466560213	
220	MAPKAPK3	Non-hit	-	-	48.63333333	15.85582017	-0.631226779	
630	MAPKAPK5	Non-hit	-	-	78.44333333	3.740806508	0.994537507	
87	MARK1	Non-hit	-	-	35.52666667	2.93227443	-1.346032233	
341	MARK2	Non-hit	-	-	57.89666667	8.927151468	-0.126027299	
320	MARK3	Non-hit	-	-	56.23666667	3.357325324	-0.216559628	
550	MARK4	Non-hit	-	-	71.85666667	11.1873783	0.635316862	
555	MAST2	Non-hit	-	-	72.28666667	8.065391084	0.658768007	
160	MAST3	Non-hit	-	-	43.64666667	1.491118149	-0.903187349	
232	MASTL	Non-hit	-	-	49.29666667	7.49822868	-0.595050206	
5	MATK	Strong hit	NM_002378	megakaryocyte-a	15.01333333	4.632022596	-2.464779121	3
639	MBIP	Non-hit	-	-	78.90666667	1.284730841	1.019806571	
745	MELK	Non-hit	-	-	88.85333333	5.114541361	1.562273375	
463	MERTK	Non-hit	-	-	66.96666667	2.956185605	0.368628255	
194	MET	Non-hit	-	-	46.24333333	3.193843035	-0.761571518	
431	MGC16169	Non-hit	-	-	64.67	3.482341167	0.243373688	
767	MGC26597	Non-hit	-	-	92.63333333	3.316961461	1.768425304	
89	MGC42105	Non-hit	-	-	35.64	12.87979814	-1.339851311	
399	MGC45428	Non-hit	-	-	61.82333333	13.31585646	0.08812347	
33	MGC4796	Weak hit	NM_032017	serine/threonine	28.58	3.586237583	-1.724886396	1
449	MGC5601	Non-hit	-	-	65.90666667	3.414693739	0.310818455	
336	MGC8407	Non-hit	-	-	57.57333333	1.45507159	-0.143661106	
709	MIDORI	Non-hit	-	-	84.36333333	0.568536132	1.317399787	
148	MINK	Non-hit	-	-	42.17333333	12.69645751	-0.983539335	
39	MKNK1	Weak hit	NM_003684	map kinase inter	29.14666667	9.872215219	-1.693981785	1
528	MKNK2	Non-hit	-	-	70.48333333	1.037802165	0.56041863	
715	MOS	Non-hit	-	-	84.88333333	0.848783443	1.345759312	
693	MPP1	Non-hit	-	-	82.4	3.32004518	1.210324403	
406	MPP2	Non-hit	-	-	62.35333333	5.826545575	0.11702837	
696	MPP3	Non-hit	-	-	82.72333333	3.009341013	1.22795821	
572	MPZL1	Non-hit	-	-	73.41666667	4.840003444	0.720395436	
577	MRC2	Non-hit	-	-	73.65	1.065316854	0.733120863	
670	MST1R	Non-hit	-	-	80.84	3.783424375	1.125245829	
210	MST4	Non-hit	-	-	47.64666667	8.738628802	-0.685037159	
251	MUSK	Non-hit	-	-	50.99333333	5.480194644	-0.502518168	
618	MVD	Non-hit	-	-	77.32666667	4.599177463	0.933637246	
533	MVK	Non-hit	-	-	70.84	2.987892234	0.579870355	

497	MYLK	Non-hit	-	-	69.14666667	2.875001449	0.487520108	
632	MYLK2	Non-hit	-	-	78.54333333	5.73323934	0.999991262	
434	MYO3A	Non-hit	-	-	64.78	3.538092707	0.249372818	
739	MYO3B	Non-hit	-	-	88.07	2.442150692	1.519552296	
704	NAGK	Non-hit	-	-	83.45666667	2.778422814	1.267952411	
541	NBEA	Non-hit	-	-	71.48	7.700954486	0.614774386	
573	NEK1	Non-hit	-	-	73.47	5.818513556	0.723304105	
660	NEK11	Non-hit	-	-	80.10333333	5.671766333	1.085069836	
606	NEK2	Non-hit	-	-	76.27	10.42292186	0.876009237	
303	NEK3	Non-hit	-	-	54.86333333	13.86968036	-0.291457859	
122	NEK4	Non-hit	-	-	39.50333333	4.709292233	-1.129154587	
139	NEK6	Non-hit	-	-	41.25	1.576166235	-1.033895671	
131	NEK7	Non-hit	-	-	40.15666667	6.119038596	-1.093523389	
723	NEK8	Non-hit	-	-	86.07	3.467794688	1.410477201	
390	NEK9	Non-hit	-	-	61.32333333	8.738434261	0.060854696	
671	NLK	Non-hit	-	-	80.92	9.690841037	1.129608833	
344	NME1	Non-hit	-	-	58.06666667	3.796529117	-0.116755916	
561	NME2	Non-hit	-	-	72.56666667	4.449295824	0.67403852	
749	NME3	Non-hit	-	-	89.72333333	2.522941405	1.609721041	
38	NME4	Average hit	NM_005009	non-metastatic c	29.11666667	6.14861231	-1.695617912	2
276	NME5	Non-hit	-	-	53.33333333	3.095308278	-0.374900307	
583	NME6	Non-hit	-	-	74.32	4.961562254	0.76966102	
474	NME7	Non-hit	-	-	67.60333333	1.459737419	0.403350494	
450	NPR1	Non-hit	-	-	65.95	3.85591753	0.313181749	
383	NPR2	Non-hit	-	-	60.91	3.785445813	0.03831251	
705	NRBP	Non-hit	-	-	83.52333333	0.448367409	1.271588248	
452	NRG3	Non-hit	-	-	66.09333333	4.595196768	0.320998797	
662	NTRK1	Non-hit	-	-	80.23333333	0.585348899	1.092159717	
165	NTRK2	Non-hit	-	-	44.04666667	7.495747683	-0.88137233	
94	NTRK3	Non-hit	-	-	36.05666667	3.227388005	-1.317127333	
501	NYD-SP25	Non-hit	-	-	69.29333333	5.095305028	0.495518949	
557	OSR1	Non-hit	-	-	72.34666667	7.598436243	0.66204026	
40	P15RS	Average hit	NM_018170	regulation of nuc	29.17	25.762758	-1.692709243	2
2	p21cip1/waf1	Strong hit	NM_000389	cyclin-depndent	11.83666667	2.630709663	-2.63802673	3
184	PACE-1	Non-hit	-	-	45.43	4.816731257	-0.805928723	
263	PACSIN1	Non-hit	-	-	52.59333333	3.990818629	-0.415258092	
764	PAG	Non-hit	-	-	91.93	2.975415937	1.730067229	
635	PAK1	Non-hit	-	-	78.70333333	14.33451197	1.008717269	

654	PAK2	Non-hit	-	-	79.97333333	2.551907783	1.077979954		
506	PAK3	Non-hit	-	-	69.58	5.013352172	0.511153046		
41	PAK4	Average hit	NM_005884	p21(cdkn1a)-act	29.61	2.821985117	-1.668712722		2
369	PAK6	Non-hit	-	-	59.91	10.01848791	-0.016225037		
614	PAK7	Non-hit	-	-	76.94	3.169794946	0.912549394		
493	PANK1	Non-hit	-	-	68.87	2.30850601	0.472431387		
476	PANK3	Non-hit	-	-	67.81333333	0.503322296	0.414803379		
650	PANK4	Non-hit	-	-	79.53666667	8.511664545	1.054165225		
589	PAPSS1	Non-hit	-	-	74.69333333	1.487761182	0.790021704		
610	PAPSS2	Non-hit	-	-	76.38333333	4.23535516	0.882190159		
208	PASK	Non-hit	-	-	47.54333333	9.988520077	-0.690672706		
345	PCK1	Non-hit	-	-	58.09	2.375015789	-0.115483373		
186	PCK2	Non-hit	-	-	45.65666667	4.572136627	-0.793566879		
633	PCTK1	Non-hit	-	-	78.58333333	3.941082762	1.002172764		
154	PCTK2	Non-hit	-	-	43.03666667	2.314850607	-0.936455253		
388	PCTK3	Non-hit	-	-	61.22	1.942472651	0.05521915		
386	PDGFRA	Non-hit	-	-	61.14666667	4.155361998	0.05121973		
167	PDGFRB	Non-hit	-	-	44.17333333	0.939432453	-0.874464241		
284	PDK1	Non-hit	-	-	53.91666667	2.259741873	-0.343086738		
620	PDK2	Non-hit	-	-	77.41	10.53140067	0.938182041		
454	PDK3	Non-hit	-	-	66.22	4.090036675	0.327906887		
503	PDK4	Non-hit	-	-	69.38666667	3.530302159	0.50060912		
241	PDPK1	Non-hit	-	-	50.25666667	9.433012951	-0.542694161		
106	PDXK	Non-hit	-	-	36.89	9.973108843	-1.271679377		
513	PFKFB1	Non-hit	-	-	69.87666667	2.024359981	0.527332518		
741	PFKFB2	Non-hit	-	-	88.23666667	3.586716233	1.528641887		
517	PFKFB3	Non-hit	-	-	69.98	3.204855691	0.532968065		
201	PFKFB4	Non-hit	-	-	46.59333333	0.355293306	-0.742483376		
209	PFKL	Non-hit	-	-	47.60666667	5.357091873	-0.687218661		
776	PFKM	Non-hit	-	-	95.35	1.370437886	1.916585641		
487	PFKP	Non-hit	-	-	68.48333333	8.535820601	0.451343535		
88	PFTK1	Non-hit	-	-	35.57333333	13.58525058	-1.343487148		
46	PGK1	Non-hit	-	-	30.68333333	5.710449486	-1.610175754		
228	PGK2	Non-hit	-	-	49.03666667	6.163313503	-0.609229969		
144	PHKA1	Non-hit	-	-	41.72333333	1.118048896	-1.008081232		
370	PHKA2	Non-hit	-	-	59.95333333	6.406062233	-0.013861743		
352	PHKG1	Non-hit	-	-	58.93666667	11.55259855	-0.06930825		
182	PHKG2	Non-hit	-	-	45.34666667	8.551218237	-0.810473518		

156	PI4K2B	Non-hit	-	-	43.30666667	3.840473061	-0.921730115
99	PI4KII	Non-hit	-	-	36.3	3.034913508	-1.30385653
768	PIK3C2A	Non-hit	-	-	93.07666667	4.488923405	1.792603616
701	PIK3C2B	Non-hit	-	-	83.15666667	4.363672001	1.251591147
697	PIK3C2G	Non-hit	-	-	82.88333333	0.900018518	1.236684217
648	PIK3CA	Non-hit	-	-	79.43	2.620744169	1.048347887
198	PIK3CB	Non-hit	-	-	46.42333333	2.596542573	-0.751754759
299	PIK3CG	Non-hit	-	-	54.7	3.587143711	-0.300365659
375	PIK3R1	Non-hit	-	-	60.24	1.192644121	0.001772353
658	PIK3R2	Non-hit	-	-	80.06333333	7.184610869	1.082888334
79	PIK3R3	Non-hit	-	-	34.80333333	9.036538792	-1.385481059
161	PIK3R4	Non-hit	-	-	43.72666667	7.800316233	-0.898824345
609	PIK4CA	Non-hit	-	-	76.33666667	4.775775679	0.879645074
677	PIK4CB	Non-hit	-	-	81.42	3.603928412	1.156877606
310	PIM1	Non-hit	-	-	55.15333333	6.492128567	-0.275641971
700	PIM2	Non-hit	-	-	83.13666667	6.650130325	1.250500396
362	PINK1	Non-hit	-	-	59.30666667	3.765864221	-0.049129357
608	PIP5K1A	Non-hit	-	-	76.32	11.16323878	0.878736115
322	PIP5K2A	Non-hit	-	-	56.60333333	6.378058743	-0.196562527
771	PIP5K2B	Non-hit	-	-	94.07666667	2.705038509	1.847141164
164	PIP5K2C	Non-hit	-	-	44.04666667	1.420715782	-0.88137233
674	PIP5KL1	Non-hit	-	-	81.03666667	2.50998672	1.135971546
673	PITPNM3	Non-hit	-	-	80.98333333	2.092876808	1.133062877
380	PKIA	Non-hit	-	-	60.69333333	2.396441807	0.026496042
280	PKIB	Non-hit	-	-	53.71666667	2.855982026	-0.353994247
252	PKLR	Non-hit	-	-	51.05	4.521758507	-0.499427707
367	PKM2	Non-hit	-	-	59.76666667	3.168743179	-0.024042086
507	PKMYT1	Non-hit	-	-	69.63	6.875587248	0.513879923
522	PKN3	Non-hit	-	-	70.16333333	8.755811403	0.542966615
83	PLK1	Non-hit	-	-	34.99333333	12.50160923	-1.375118925
108	PLK2	Non-hit	-	-	37.01	5.262480404	-1.265134871
307	PLK3	Non-hit	-	-	55.05666667	11.23570351	-0.280913934
289	PLK4	Non-hit	-	-	54.14	6.797242088	-0.330906685
407	PMVK	Non-hit	-	-	62.35333333	9.049885819	0.11702837
748	PNKP	Non-hit	-	-	89.54666667	2.047933918	1.600086074
505	PPP1R1B	Non-hit	-	-	69.55333333	4.796502198	0.509698711
711	PPP2CA	Non-hit	-	-	84.83333333	4.112983508	1.343032435
445	PPP2CB	Non-hit	-	-	65.43666667	8.901108545	0.285185808

202	PPP4C	Non-hit	-	-	46.98666667	3.053102247	-0.721031941	
146	PRKAA1	Non-hit	-	-	41.85333333	7.591523782	-1.00099135	
292	PRKAA2	Non-hit	-	-	54.34666667	9.969685719	-0.319635592	
73	PRKACA	Non-hit	-	-	34.42	4.117912092	-1.406387119	
151	PRKACB	Non-hit	-	-	42.57	9.437695693	-0.961906108	
22	PRKACG	Strong hit	NM_002732	protein kinase, c	23.44666667	3.380078895	-2.004845805	3
290	PRKAG1	Non-hit	-	-	54.15666667	1.423036659	-0.329997726	
775	PRKAG3	Non-hit	-	-	95.34	2.892213685	1.916040265	
540	PRKAR1A	Non-hit	-	-	71.46666667	1.510110371	0.614047218	
509	PRKAR2A	Non-hit	-	-	69.7	2.314497786	0.517697551	
428	PRKAR2B	Non-hit	-	-	64.20333333	2.609297479	0.217922833	
486	PRKCA	Non-hit	-	-	68.48	10.28418203	0.451161744	
130	PRKCABP	Non-hit	-	-	40.06666667	6.129488831	-1.098431768	
451	PRKCB1	Non-hit	-	-	65.99333333	15.29748127	0.315545043	
518	PRKCD	Non-hit	-	-	70.05333333	5.430776495	0.536967485	
544	PRKCE	Non-hit	-	-	71.73	8.786284767	0.628408772	
191	PRKCG	Non-hit	-	-	46.04666667	4.548761736	-0.772297235	
637	PRKCH	Non-hit	-	-	78.74333333	17.28686303	1.010898771	
389	PRKCI	Non-hit	-	-	61.23333333	2.904226805	0.055946317	
604	PRKCL1	Non-hit	-	-	76.12	1.390827092	0.867828605	
519	PRKCL2	Non-hit	-	-	70.08666667	16.39651284	0.538785403	
227	PRKCM	Non-hit	-	-	49.00333333	2.283995038	-0.611047887	
262	PRKCN	Non-hit	-	-	52.46333333	5.378274197	-0.422347973	
240	PRKCQ	Non-hit	-	-	50.05	9.015026345	-0.553965254	
737	PRKCSH	Non-hit	-	-	87.79333333	2.71941783	1.504463575	
132	PRKCZ	Non-hit	-	-	40.17	2.125158818	-1.092796222	
56	PRKD2	Non-hit	-	-	32.17	12.18827715	-1.529096601	
761	PRKDC	Non-hit	-	-	91.22	3.611744731	1.69134557	
115	PRKG1	Non-hit	-	-	38.42333333	7.975100835	-1.188055138	
117	PRKG2	Non-hit	-	-	38.65	3.558187741	-1.175693294	
411	PRKR	Non-hit	-	-	62.76333333	5.34642248	0.139388765	
358	PRKRA	Non-hit	-	-	59.08333333	3.309325208	-0.06130941	
549	PRKWNK1	Non-hit	-	-	71.81666667	4.397923753	0.63313536	
95	PRKWNK2	Non-hit	-	-	36.10666667	3.984499132	-1.314400456	
523	PRKWNK3	Non-hit	-	-	70.32666667	2.558228554	0.551874414	
28	PRKWNK4	Weak hit	NM_032387	wnk lysine defici	26.88	1.055888252	-1.817600226	1
717	PRKX	Non-hit	-	-	85.14333333	2.790023895	1.359939074	
261	PRKY	Non-hit	-	-	52.38666667	6.35512654	-0.426529185	

656	PRPF4B	Non-hit	-	-	80.02333333	10.60526442	1.080706832	
6	PRPK	Strong hit	NM_033550	tp53 regulating k	16.01	2.275675724	-2.410423366	3
4	PRPS1	Strong hit	NM_002764	phosphoribosyl p	14.95	1.007769815	-2.468233166	3
661	PRPS1L1	Non-hit	-	-	80.14	4.09	1.087069546	
275	PRPS2	Non-hit	-	-	53.24	6.803682826	-0.379990478	
622	PRPSAP1	Non-hit	-	-	77.75333333	5.211356189	0.956906599	
672	PRPSAP2	Non-hit	-	-	80.96	1.084297007	1.131790334	
188	PSKH1	Non-hit	-	-	45.86666667	17.45317259	-0.782113994	
301	PSKH2	Non-hit	-	-	54.75333333	7.268131351	-0.29745699	
668	PTK2	Non-hit	-	-	80.61666667	2.145701129	1.113065777	
343	PTK2B	Non-hit	-	-	57.99333333	3.211795967	-0.120755336	
13	PTK6	Weak hit	NM_005975	ptk6 protein tyro	20.52666667	1.826535883	-2.164095443	1
317	PTK7	Non-hit	-	-	55.90666667	4.281732515	-0.234557018	
37	PTK9	Non-hit	-	-	28.99333333	0.760548048	-1.702344209	
11	PTK9L	Average hit	NM_007284	prk9l protein tyrc	19.17666667	3.056948369	-2.237721132	2
592	PTPN5	Non-hit	-	-	74.87666667	9.483967173	0.800020255	
229	PTPRG	Non-hit	-	-	49.16333333	6.979916427	-0.602321879	
400	PTPRJ	Non-hit	-	-	61.83	2.305666932	0.088487054	
758	PTPRR	Non-hit	-	-	90.59333333	7.037715065	1.657168707	
401	PTPRT	Non-hit	-	-	61.89	3.678681829	0.091759307	
575	PXK	Non-hit	-	-	73.6	1.768813161	0.730393986	
308	PYCS	Non-hit	-	-	55.08333333	3.807838407	-0.279459599	
640	RAC1	Non-hit	-	-	78.96	6.576982591	1.02271524	
376	RAF1	Non-hit	-	-	60.27333333	2.628808349	0.003590272	
141	RAGE	Non-hit	-	-	41.39333333	8.668969566	-1.026078622	
663	RAPGEF3	Non-hit	-	-	80.23666667	6.591132932	1.092341509	
235	RAPGEF4	Non-hit	-	-	49.45666667	7.360056612	-0.586324199	
676	RASGRF2	Non-hit	-	-	81.35333333	7.896336703	1.15324177	
174	RBKS	Non-hit	-	-	44.94	6.881591386	-0.832652121	
96	RET	Non-hit	-	-	36.16333333	2.581498273	-1.311309995	
347	RFK	Non-hit	-	-	58.18	0.664605146	-0.110574994	
391	RFP	Non-hit	-	-	61.42333333	2.334958958	0.066308451	
430	RIOK1	Non-hit	-	-	64.45333333	14.33584435	0.23155722	
168	RIOK3	Non-hit	-	-	44.24666667	2.11095555	-0.87046482	
304	RIPK1	Non-hit	-	-	54.90333333	5.457374216	-0.289276358	
753	RIPK2	Non-hit	-	-	90.1	2.374257779	1.630263517	
752	RIPK3	Non-hit	-	-	90.04666667	1.212284345	1.627354848	
357	RNASEL	Non-hit	-	-	59.07333333	6.155983539	-0.061854785	

404	ROCK1	Non-hit	-	-	62.07666667	0.765005447	0.101939649	
570	ROCK2	Non-hit	-	-	73.35	9.365452472	0.716759599	
224	ROR1	Non-hit	-	-	48.91666667	3.097811055	-0.615774474	
107	ROR2	Non-hit	-	-	36.99666667	1.081911888	-1.265862039	
394	ROS1	Non-hit	-	-	61.58666667	11.91521856	0.075216251	
649	RP2	Non-hit	-	-	79.43666667	1.873241398	1.048711471	
529	RPS6KA1	Non-hit	-	-	70.54666667	5.865341706	0.563872675	
588	RPS6KA2	Non-hit	-	-	74.58666667	15.45845184	0.784204366	
77	RPS6KA3	Non-hit	-	-	34.54333333	7.225242787	-1.399660822	
414	RPS6KA4	Non-hit	-	-	62.96333333	4.50551144	0.150296274	
765	RPS6KA5	Non-hit	-	-	92.06333333	4.850920875	1.737338902	
371	RPS6KA6	Non-hit	-	-	59.98666667	16.91500616	-0.012043825	
731	RPS6KB1	Non-hit	-	-	86.75333333	4.549750909	1.447744525	
29	RPS6KB2	Average hit	NM_003952	ribosomal protei	26.9	3.464289249	-1.816509475	2
582	RPS6KC1	Non-hit	-	-	74.2	4.753493452	0.763116514	
461	RPS6KL1	Non-hit	-	-	66.83333333	10.02521488	0.361356582	
270	RYK	Non-hit	-	-	52.92666667	4.225071991	-0.397078909	
381	SAST	Non-hit	-	-	60.70333333	11.41513177	0.027041417	
651	SCAP1	Non-hit	-	-	79.67666667	4.017291791	1.061800482	
76	SCYL1	Non-hit	-	-	34.50333333	1.67622592	-1.401842323	
581	SEPHS1	Non-hit	-	-	74.06333333	4.473257575	0.75566305	
189	SEPHS2	Non-hit	-	-	45.94333333	2.350581488	-0.777932782	
199	SGK	Non-hit	-	-	46.48	4.101426581	-0.748664298	
607	SGK2	Non-hit	-	-	76.30666667	6.847585949	0.878008947	
392	SGKL	Non-hit	-	-	61.51666667	8.327090328	0.071398622	
612	SHC1	Non-hit	-	-	76.42666667	1.420434206	0.884553453	
267	SIK2	Non-hit	-	-	52.87	10.15172399	-0.40016937	
	siRNA target gei	Hit definition	Hit Accession Nı	Full Name of Hit	Average	Standard Deviat	Z-score	
455	SLK	Non-hit	-	-	66.25	3.47667945	0.329543013	
321	SMAD7	Non-hit	-	-	56.59	4.79070976	-0.197289694	
467	SMG1	Non-hit	-	-	67.17666667	9.719682779	0.38008114	
319	SNARK	Non-hit	-	-	56.15	6.552198104	-0.221286215	
110	SNF1LK	Non-hit	-	-	37.31	1.914445089	-1.248773607	
412	SNRK	Non-hit	-	-	62.81666667	6.503632318	0.142297434	
595	SOCS1	Non-hit	-	-	75.42	2.999266577	0.829652322	
532	SOCS5	Non-hit	-	-	70.73333333	3.455580028	0.574053017	
774	SPA17	Non-hit	-	-	95.27	3.313668662	1.912222637	
591	SPEC2	Non-hit	-	-	74.86	6.598909001	0.799111296	

699	SPHK1	Non-hit	-	-	82.92666667	12.4747839	1.239047511	
12	SPHK2	Strong hit	NM_020126	sphingosine kina	19.45333333	2.310418433	-2.222632411	3
567	SQSTM1	Non-hit	-	-	73.19666667	5.218010477	0.708397175	
169	SRC	Non-hit	-	-	44.32	6.219075494	-0.8664654	
721	SRMS	Non-hit	-	-	85.66	0.221133444	1.388116807	
479	SRPK1	Non-hit	-	-	68.11	9.690515982	0.430982851	
576	SRPK2	Non-hit	-	-	73.64666667	10.2451273	0.732939072	
457	SSTK	Non-hit	-	-	66.67	11.18540567	0.352448783	
435	STK10	Non-hit	-	-	64.78333333	7.77988646	0.24955461	
311	STK11	Non-hit	-	-	55.21333333	5.832498035	-0.272369718	
18	STK16	Average hit	NM_003691	serine/threonine	22.06666667	2.743799069	-2.080107621	2
85	STK17A	Non-hit	-	-	35.16666667	9.305059556	-1.36566575	
397	STK17B	Non-hit	-	-	61.81	1.126410227	0.087396303	
763	STK19	Non-hit	-	-	91.71	2.658589852	1.718068968	
128	STK22B	Non-hit	-	-	39.82333333	6.934942922	-1.111702572	
408	STK22C	Non-hit	-	-	62.35666667	8.076102608	0.117210162	
647	STK22D	Non-hit	-	-	79.40666667	9.330028582	1.047075344	
64	STK23	Weak hit	NM_014370	serine/threonine	33.40333333	5.16153401	-1.461833625	1
492	STK24	Non-hit	-	-	68.85333333	14.12555958	0.471522428	
192	STK25	Non-hit	-	-	46.09	0.834326075	-0.769933941	
24	STK29	Average hit	NM_003957	br serine/threoni	24.37333333	6.663575117	-1.954307678	2
437	STK3	Non-hit	-	-	64.91666667	0.410406303	0.256826283	
419	STK31	Non-hit	-	-	63.36333333	6.744518762	0.172111293	
173	STK32A	Non-hit	-	-	44.94	3.75004	-0.832652121	
264	STK32B	Non-hit	-	-	52.61333333	1.88505526	-0.414167341	
587	STK32C	Non-hit	-	-	74.56666667	6.565366199	0.783113615	
239	STK33	Non-hit	-	-	50.04	6.818438238	-0.554510629	
770	STK35	Non-hit	-	-	94.04	0.918313672	1.845141454	
652	STK36	Non-hit	-	-	79.68	4.543203715	1.061982274	
559	STK38	Non-hit	-	-	72.42333333	16.16341032	0.666221472	
710	STK38L	Non-hit	-	-	84.79	10.14813776	1.340669141	
339	STK39	Non-hit	-	-	57.73333333	6.36767095	-0.134935099	
44	STK4	Weak hit	NM_006282	serine/threonine	29.94	8.711412055	-1.650715331	1
659	STK6	Non-hit	-	-	80.08666667	2.963786992	1.084160876	
459	STYK1	Non-hit	-	-	66.75333333	2.390111573	0.356993579	
556	SYK	Non-hit	-	-	72.32	4.645557017	0.660585925	
515	TAF1	Non-hit	-	-	69.93666667	0.803264174	0.530604771	
682	TAF1L	Non-hit	-	-	81.65	4.543996039	1.169421242	

382	TAO1	Non-hit	-	-	60.76666667	9.144377143	0.030495462	
475	TAO1	Non-hit	-	-	67.71666667	7.429416756	0.409531416	
636	TBK1	Non-hit	-	-	78.74	0.765441049	1.010716979	
7	TEC	Average hit	NM_003215	tec protein tyros	17.32666667	2.011475412	-2.338615595	2
116	TEK	Non-hit	-	-	38.43666667	8.121947632	-1.187327971	
253	TESK1	Non-hit	-	-	51.10333333	2.524684799	-0.496519037	
443	TESK2	Non-hit	-	-	65.31333333	2.679745013	0.27845951	
417	TEX14	Non-hit	-	-	63.13	1.590880259	0.159385865	
19	TGFBR1	Average hit	NM_004612	transforming gro	22.18333333	3.815983927	-2.073744907	2
72	TGFBR2	Non-hit	-	-	34.40333333	0.863963734	-1.407296078	
495	THNSL1	Non-hit	-	-	69.03666667	3.487195626	0.481520978	
396	TIE	Non-hit	-	-	61.7	9.379216385	0.081397173	
402	TJP2	Non-hit	-	-	61.94333333	2.249051652	0.094667976	
464	TK1	Non-hit	-	-	67.04	8.566930606	0.372627675	
433	TK2	Non-hit	-	-	64.76666667	6.332308689	0.248645651	
265	TLK1	Non-hit	-	-	52.71333333	15.19877078	-0.408713586	
543	TLK2	Non-hit	-	-	71.71	3.260184044	0.627318022	
446	TLR1	Non-hit	-	-	65.45333333	11.17573413	0.286094767	
720	TLR3	Non-hit	-	-	85.39333333	3.400725413	1.373573461	
779	TLR4	Non-hit	-	-	96.70666667	1.317510278	1.990574913	
245	TLR6	Non-hit	-	-	50.34666667	4.711691133	-0.537785782	
231	TNFRSF10A	Non-hit	-	-	49.22666667	2.991092331	-0.598867835	
742	TNIK	Non-hit	-	-	88.31333333	7.831170623	1.532823099	
60	TNK1	Non-hit	-	-	32.94666667	2.350241122	-1.486739105	
52	TNNI3K	Non-hit	-	-	31.65	4.58670906	-1.557456125	
315	TOPK	Non-hit	-	-	55.75333333	2.41630572	-0.242919442	
751	TPK1	Non-hit	-	-	89.92666667	1.595692117	1.620810342	
728	TRAD	Non-hit	-	-	86.44666667	2.44532888	1.431019678	
147	TRIB1	Non-hit	-	-	42.11333333	5.428778254	-0.986811588	
574	TRIB2	Non-hit	-	-	73.51	17.178146	0.725485607	
283	TRIB3	Non-hit	-	-	53.91666667	4.68788154	-0.343086738	
646	TRIM	Non-hit	-	-	79.17	2.755013612	1.034168125	
176	TRIO	Non-hit	-	-	45.11	11.56517618	-0.823380738	
118	TRPM6	Non-hit	-	-	38.9	0.182482876	-1.162058907	
531	TRPM7	Non-hit	-	-	70.70333333	2.96123848	0.572416891	
373	TSKS	Non-hit	-	-	60.02666667	5.085787386	-0.009862323	
248	TTBK1	Non-hit	-	-	50.83333333	6.54839166	-0.511244175	
643	TTBK2	Non-hit	-	-	79.05333333	4.008058549	1.027805411	

726	TTK	Non-hit	-	-	86.17333333	2.985235892	1.416112748	
121	TTN	Non-hit	-	-	39.46666667	1.947160326	-1.131154297	
478	TXK	Non-hit	-	-	67.88	5.547440851	0.418439215	
363	TXNDC3	Non-hit	-	-	59.31	4.959475779	-0.048947566	
135	TYK2	Non-hit	-	-	40.77666667	3.371977659	-1.05971011	
510	TYRO3	Non-hit	-	-	69.75333333	7.409590632	0.520606221	
138	UCK1	Non-hit	-	-	41.11333333	7.794140962	-1.041349135	
361	UGP2	Non-hit	-	-	59.29	7.745217879	-0.050038317	
10	ULK1	Strong hit	NM_003565	unc-51-like kinas	19.17	3.139761137	-2.238084716	3
442	ULK2	Non-hit	-	-	65.13	2.771281292	0.26846096	
351	UMP-CMPK	Non-hit	-	-	58.66	4.212730706	-0.084396971	
481	UMPK	Non-hit	-	-	68.28	7.174210758	0.440254234	
49	URKL1	Non-hit	-	-	31.22	4.298464842	-1.580907271	
66	VRK1	Non-hit	-	-	33.66	6.024707462	-1.447835655	
586	VRK2	Non-hit	-	-	74.43	3.039950657	0.77566015	
511	VRK3	Non-hit	-	-	69.8	0.206639783	0.523151306	
605	WEE1	Non-hit	-	-	76.15666667	2.148588684	0.869828315	
179	WIF1	Non-hit	-	-	45.19666667	6.927945823	-0.81865415	
489	XYLB	Non-hit	-	-	68.59	4.590130717	0.457160874	
59	YES1	Non-hit	-	-	32.5	2.600057692	-1.51109921	
688	YWHAH	Non-hit	-	-	81.84	0.592283716	1.179783376	
747	YWHAQ	Non-hit	-	-	89.38	4.313548423	1.590996483	
14	ZAK	Strong hit	NM_133646	sterile alpha mot	21.10333333	2.867792415	-2.132645458	3
20	ZAP70	Average hit	NM_001079	zeta-chain (tcr) a	23.14333333	4.652680231	-2.021388861	2

Hit Definitions
